# In-silico study of active phytochemicals and molecular mechanism of *Hydrocotyle javanica* Thunb.in treating MDR enterobacterial infection

**DOI:** 10.1186/s40643-026-01078-5

**Published:** 2026-08-20

**Authors:** Debasmita Paul, Megha Ghosh, Manab Mandal

**Affiliations:** 1 Department of Botany, Jogamaya Devi College, Kolkata, 700026 West Bengal India; 2 Department of Botany, Dukhulal Nibaran Chandra College, Murshidabad, 742201 India

**Keywords:** Enterobacterial infection, Stigmasterol, MDR, *Hydrocotyle javanica*, Network pharmacology, Molecular dynamics simulation

## Abstract

Enterobacterial infections being severe, with a high mortality rate, particularly affecting ICU patients, and 80% newborns. These infections have multifactorial multi-drug resistance (MDR) mechanisms, which frequently causes current antibiotic therapies to fail, mortality rate remains high, and creating an urgent need for alternative multi-targeted therapies, such as plant-derived compounds to restore clinical effectiveness. This study aims to discover novel anti enteric compounds in *Hydrocotyle javanica* Thunb. *(H. javanica)* belongs to Apiaceae family and understanding their interaction mechanism with enterobacterial infection targeted genes, using network pharmacology with in-silico docking and molecular dynamics simulation approaches. Four bioactive compounds as tetracosanoic acid, alpha-Amyrenyl acetate, stigmasterol glucoside, stigmasterol were identified as potential therapeutic agents. Most of the compounds exhibited favourable pharmacokinetic properties, complying with Lipinski’s rule, with high bioavailability score 0.85 and non-toxic profiles. In-silico antibacterial prediction indicated both bacterial and bacteriostatic activities. A total of 53 common targets were identified with network analysis revealing key hub genes including HSD11B1, PTGS2,FDFT1,CYPHA1, and AKR1C2. Functional enrichment analysis showed significant involvement in immune and inflammatory pathways, particularly calcium signaling, MAPK signaling and reactive oxygen species related pathways. The docking result showed highest binding affinity, with stigmasterol showing highest score (-10.7 kcal/mol). A 100 ns molecular dynamics simulation further confirmed the stability of the stigmasterol-protein complex, with stable RMSD values 1–2 Å, low RMSF fluctuations and consistent hydrogen bonding, indicating sustained structural integrity. The present study highlights that phytocompounds from *Hydrocotyle javanica* exhibit significant binding affinity toward key enterobacterial targets, along with favorable ADME and toxicity profiles. These findings suggest their potential as promising lead molecules for anti-enterobacterial drug development, warranting further experimental validation.

**Graphical Abstract:**

**Figure:**
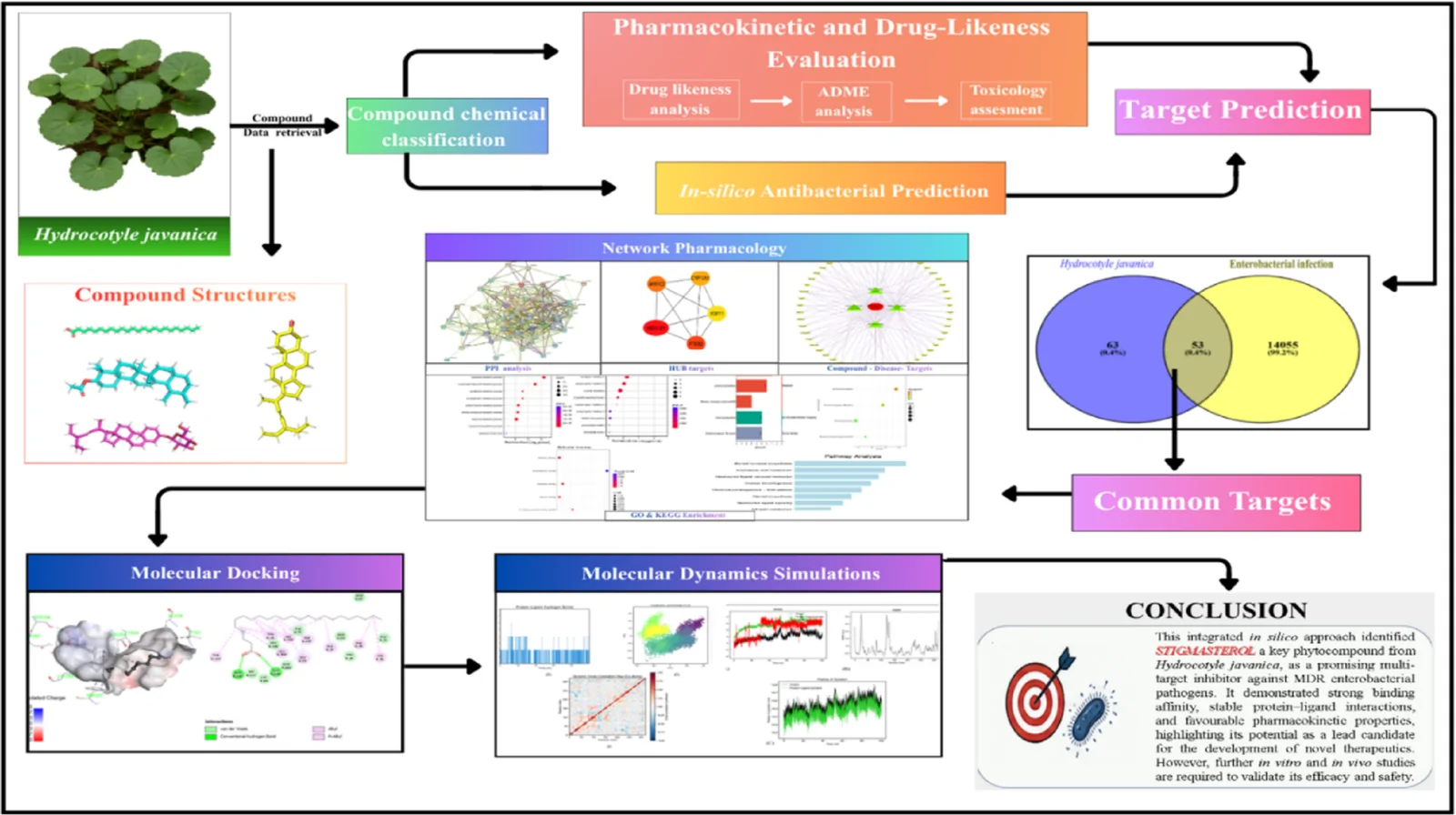

**Supplementary Information:**

The online version contains supplementary material available at 10.1186/s40643-026-01078-5.

## Introduction

Enterobacterial infection showing high clinical importance due to severe outcomes and mortality rates among neonates and immunocompromised patients. Neonatal *Cronobacter* meningitis shows 40–80% mortality (LAI 2001; Salimiyan Rizi et al. 2020), while *Enterobacter bacteraemia* showing 24.6–34.7% mortality. Clinically important species include *Cronobacter sakazakii*, *Enterobacter cloacae(E. cloacae)*,* Klebsiella aerogenes*,* Pantoea agglomerans* (Salimiyan Rizi et al. 2020). These organisms act as opportunistic pathogens and are major causes of nosocomial infections, particularly in ICUs, emergency wards, neonatal units, and patients with invasive devices. Their pathogenicity is driven by multiple virulence factors, including adhesins, LPS capsule, biofilm formation, enterotoxins, hemolysins, pore forming toxins, siderophores, and their ability to survive inside macrophage (Anju et al. 2020). As a result, enterobacter infections lead to inflammation, SIRS, sepsis, and multiple organ infections, affecting the bloodstream, respiratory tract, urinary tract, wounds, brain, skin, and bone. These enterobacter pathogens are strongly associated with multidrug resistance. The major mechanism is β-lactamase production, including AmpC β-lactamases, broad-spectrum β-lactamases, plasmid-mediated β-lactamases, and in some strains, carbapenemase (Anju et al. 2020). This results in resistance to aminopenicillins, amoxicillin clavulanate, first generation cephalosporins, cefoxitin, broadspectrum cephalosporins, carbapenems, and multiple other classes. Efflux pumps, porin mutations, biofilm formation, and membrane impermeability further enhance MDR (Mondal et al. 2021). *Enterobacter asburiae* BDW1M3 shows resistance to beta-lactams, glycopeptides, phosphonic acids, tetracyclines, and fluoroquinolones, and carries resistance genes such as blaACT-2, fosA2, qnrE2, van clusters, and multiple efflux systems (Gaston 1988). Despite weak biofilm formation, it persists in wounds and enhances antibiotic tolerance, confirming the failure of conventional antibiotics (Bassetti et al. 2015).

This rising resistance highlights an urgent research gap and the need for alternative therapeutic pathways (Varshan and Namasivayam 2025) as current therapeutic approaches largely focus on single -target interventions, which are incapable against complex resistance pathways and also, there is a lack of strategies targeting non-carbapenemase based carbapenem resistance (Bassetti et al. 2015), biofilm-specific survival, metal resistance genes, and prophage mediated AMR transfer. Plant-based herbal products(Raj et al. 2026) therapeutics present a promising alternative because they can target multiple pathways simultaneously, inhibit efflux pumps, disrupt the membranes, block virulence factors, and act as anti-biofilm agents. They come with safety, cheap and are widely available with fewer side effects. *H. javanica* commonly known as Pegaga gajah or Java pennywort, belonging to the Apiaceae family (Jusoh et al.), is traditionally used as an aperient, tonic, stimulant, diuretic, blood purifier, and is applied for skin diseases, stomach ulcers, urinary disorders, digestive issues, dysentery, asthma, eye infections, and wound healing (Mandal et al. 2017). It contains sesquiterpenes, fatty acids, terpenes, ketones, flavonoids, phenolics, cardiac glycosides, saponins, and triterpenoids (Abiraamavalli et al. 2026; Mandal et al. 2020), with no reported hepatotoxicity. The plant exhibits strong antioxidant (Mandal et al. 2016) activity and significant antibacterial effects against both Gram-positive and Gram-negative pathogens, including *Bacillus cereus*,* B. subtilis*,* E. coli*,* E. faecalis*,* L. monocytogenes*,* Salmonella typhimurium*,* S. mutans*,* and Pseudomonas aeruginosa.* Its antibacterial action is linked to alkaloids, phenols, flavonoids, monoterpenes, terpenoids, and phenolic monoterpenoid thymol, which disrupt bacterial membranes, increase permeability, cause leakage, inhibit respiration, and damage cell walls (Misra et al. 2020, 2022; Priyanka and Namasivayam 2026). Phenolic compounds such as p-cresol and iso-indole alkaloids also contribute by inhibiting nucleic acid synthesis. However, there is a lack of thorough research investigating the in-silico antibacterial capabilities of *H. javanica* through systematic network pharmacology methods in relation to enterobacterial infections. Furthermore, the specific molecular processes and pathways by which this plant produces its effects are not yet well understood.

Phytocompounds or herbal therapies exert therapeutic effects through multicomponent (Mandal et al. 2020; Misra et al. 2018) and multi target mechanism and characterized by structural diversity (Misra et al. 2017; Sivasuriyan et al. 2026) making them promising candidate for complex disease like bacterial infection (Misra et al. 2022; Varshan et al. 2026). In-silico network pharmacology a comprehensive computational analysis(Gupta et al.) to extract insights from massive high dimensional biological resources allow researchers to shift from “one target, one drug” to system based multitarget strategies (Berger and Iyengar 2009), additionally it enables the prediction of synergistic drug combinations through algorithms and facilitates the repurposing of existing medications by semantically linking drug actions to novel disease mechanisms bypassing expense, in-silico approach also predicts adverse events and off targets effects before clinical testing integrate allowing rapid target identification thus compound-target-pathway relationship offers a systematic strategy to unravel the therapeutic mechanisms of plant derived phytochemicals.

The present study applied a comprehensive network pharmacology approach combining in-silico antibacterial prediction and molecular docking followed by molecular dynamics simulation to estimate the antibacterial potential of *H. javanica* against enterobacterial pathogenic infections. Computational approaches and revealing pharmacokinetic properties profiling have become valuable tools in identifying potential therapeutic compounds from plant sources (Vedhamani et al. 2025). To best of our knowledge this is the first time comprehensive study to explore in-silico antibacterial potential of *H. javanica* through systematic network pharmacology approaches against enterobacterial infection.

## Materials and methods

### Compounds retrieval and data set preparation

Phytoconstituents and bioactive compounds were retrieved from *H. javanica* using a systematic approach through the published, curated database Indian Medicinal Plants, Phytochemistry and Therapeutics (IMPPAT) (https://cb.imsc.res.in/imppat). Compounds SMILES (Simplified Molecular Input Line Entry System) and their PubChem identifier, molecular formula (MF) and 2D,3D structures obtained from PubChem (https://pubchem.0ncbi.nlm.nih.gov/) database for further computational analysis.

### Classification of compounds

Selected compounds were evaluated to chemical classification (Choudhary and Singh 2018) with tool method “ClassyFire” (Djoumbou Feunang et al. 2016). These results were mapped using hierarchical clustering toolbox from ChemMine tools (Backman et al. 2011) with atom pair descriptor and Tanimoto coefficient. This hierarchical clustering output was visualized using Interactive Tree of Life (https://itol.embl.de/) (iTOL), with compounds labeled with unique identifiers and compound classes are represented using color strips where structural dissimilarity indicated by branch length.

### Drug-likeness evaluation

The drug-likeness properties like physicochemical properties including molecular weight (MW < 500), lipophilicity (LogP), topological polar surface area (TPSA), hydrogen bond donor (HBD < 5), hydrogen bond acceptor (HBA < 10) and drug-likeness score of the compounds screened out from Molsoft L.L.C (https://molsoft.com/mprop/) webtool using SMILES characters.

### **Compounds ADME (absorption**,** distribution**,** metabolism**,** excretion) analysis**

Pharmacokinetic (ADME) properties were evaluated by using SwissADME (https://www.swissadme.ch/index.php) based on SMILES input. Fundamental pharmacokinetic properties including gastrointestinal (GI) absorption and bioavailability, blood brain barrier (BBB) permeability, p-glycoprotein (P-gp) substrate, cytochrome P450 enzyme inhibition (CYP1A2, CYP2C9, C YP2D6, CYP3A4), Lipinski’s rule, and bio-availability score were predicted.

### Toxicity prediction

In-silico toxicity profiling of phytocompounds was conducted using ProTox-II (https://tox-new.charite.de/protox_II/). Organ-specific toxicities (hepatotoxicity, nephrotoxicity), and toxicity endpoints (immunotoxicity, mutagenicity, and cytotoxicity and reproductive toxicity) were predicted. Also, estimating their toxicity classes and LD50 values in mg/kg body weight. The LD50 represents the median lethal dose, indicating the amount at which 50% of test subjects are adversely affected upon exposure to a compound.

### Anti-bacterial prediction of compounds

The in-silico prediction of antibacterial potential (Rajan et al. 2025) compounds evaluated through antiBac-Pred software of Way2Drug server (https://way2drug.com/antibac/). It predicts the ability of a chemical compound to inhibit bacterial strain at minimum inhibitory concentration below 10,000 nM, based on their structural feature similarity with existing models implemented in PASS software (Lagunin et al. 2000).

### Compound-disease target prediction

The potential target of selected four biologically active phytochemicals were predicted through the computational tool Swiss Target Prediction (https://www.swisstargetprediction.ch/) using the canonical SMILES character of each selected compound was run in the tool and organism selected as *Homo sapiens*. Prediction done using ligand-based approach, based on similarity between query molecule and known ligand of large collection of a protein target. The selected disease targets were retrieved by using the term “Enterobacterial infection” from GeneCards database (https://www.genecards.org/) and CTD (Comparative Toxicogenomics Database) (https://ctdbase.org/) (Davis et al. 2021). Duplicate entries were removed to maintain consistency among the data.

### Identifying common targets

To identify the common targets the disease targets and compounds target were intersected using the Venny 2.1 (https://bioinfogp.cnb.csic.es/tools/venny/). These intersecting common targets were identified as therapeutic targets of the plant compounds against the enterobacterial infection. These therapeutic targets were used for further protein-protein interaction, network construction, and functional enrichment analysis.

### Protein-protein interaction (PPI) analysis

Common targets of the phytochemicals and disease targets were uploaded to the STRING (Search Tool for the Retrieval of Interacting Genes/proteins) (https://string-db.org/). The organism set to *Homo sapiens* and confidence score set low to avoid any isolated nodes were to focus on core targets and other parameters set to default and the PPI network created.

### Network construction and hub-target identification

For network building and additional analysis, PPI network data was exported from the STRING database into Cytoscape (Kohl et al. 2010) software in TSV format and a network created with potential targets applying circular hierarchical layout, also a compound-target-infection network constructed to visualize the relationship between phytochemicals and predicted targets and infection. To identify the core targets, the hub genes analysis was done using the CytoHubba (Chin et al. 2014) plug-in in Cytoscape, using a topological algorithm, as degree analysis, selecting nodes.

### Gene ontology (GO) and kyoto encyclopaedia of genes and genomes (KEGG) pathway enrichment analysis

The common targets derived from the compound-disease targets analysis were used for GO and KEGG enrichment analysis. Web tool DAVID (https://davidbioinformatics.nih.gov/), which provides a full suite of functional annotation tools to help to interpret the biological significance of large gene datasets was used for the enrichment analysis. The ontologies and enriched pathways were executed by uploading the gene list, selecting *Homo sapiens* as the species, choosing the official gene symbol as the identifier, and using the functional annotation tool to predict the biological process (BP), cellular component (CC) and molecular function (MF) lists. KEGG is a collection of manually drawn pathway maps that depict molecular interaction and reaction networks, key signaling pathways related to these common targets identified with an adjusted p-value (≤ 0.05) were deemed statistically significant, and those containing hub target proteins with biological relevance were selected for further visualization and analysis. SR Bioinformatics tool (https://www.bioinformatics.com.cn/en) used to visualize top ontologies (Madikyzy et al. 2022).

### Molecular docking

Molecular docking was performed to predict the ligand as the plant’s *H. javanica* four biologically active phytochemicals and top pathways gene targets as receptor, binding affinity, and key interaction mechanism (Pal et al. 2025). The three-dimensional (3D) conformer of selected phytocompounds collected from PubChem (https://pubchem.ncbi.nlm.nih.gov/) database as Spatial Data File (SDF) file format and compound lacking 3D structure in PubChem database, the 2D structure converted into 3D structure using MolView (https://app.molview.com/) software. The crystal structure of the receptor proteins collected from the RCSB Protein Data Bank (https://www.rcsb.org/), it collected by filtering some parameters as organism set to *Homo sapiens* resolution set to minimum 1.5-2.0 and selecting crystal structure and downloaded from the database as PDB format. The protein molecules were cleaned by removing their co-crystalized ligands, water molecule, and hydrogen-bonding residues involved in the ligand interaction were identified to ensure accurate binding analysis using the SwissPDBViewer software.Molecular docking was performed using AutoDock Vina in PyRx with default exhaustiveness, and top-ranked conformations were selected based on binding energy. The ligand was energy minimized by uploading in OpenBabel a built-in tool in PyRx and converted to PDBQT format, and the receptor proteins were uploaded into the software as a macromolecule in PDBQT file format. The performed docking was visualize using BIOVIA Discovery Studio Visualizer software (Singh et al. 2026).

### Molecular dynamics simulation

MD Simulation performed using GROMACS 2023 version software, a free opensource software suite for high- performance molecular dynamics and output analysis (Misra et al. 2022). After docking, the top-ranked protein–ligand complex was simulated to assess the binding affinity of the most promising compound. Protein topology and force field parameters for simulation were prepared using CHARMM27 while the ligand parameters were sourced from SwissParam webserver. The system solvation was done using the TIP3P water model and counter ions Na + and Cl^−^ to neutralize the system. Equilibration was conducted under the NPT ensemble for 2 ns at steady temperature of 298 k. The MD simulation production performed with for 100ns the resulting trajectories used for post-simulation analysis and dynamic movement of the complex which includes Root Mean Square Deviation (RMSD), Root Mean Square Fluctuation (RMSF), Radius of Gyration (Rg), Protein-ligand hydrogen bonds, Principal Component Analysis (PCA), and Dynamic cross-correlation matrix (DCCM) of protein ligand complex were calculated.

## Results

### Phytochemicals datasets for network pharmacology analysis

A total four phytochemicals tetracosanoic acid, alpha-amyrenyl-acetate, stigmasterol glucoside, stigmasterol retrieve from the database and their SMILES, PubChem CID, Molecular formula, 3D structures represented in the (Table 1).

**Table 1:** Phytocompounds data retrieval with 3D structures

SL No.	Compound	SMILES	PubChem CID	MF	3D Structure
1	Tetracosanoic acid	CCCCCCCCCCCCCCCCCCCCCCCC(= O)O	11,197	C_24_H_48_O_2_	
2	alpha-Amyrenyl acetate	CC(= O)O[C@H]1CC[C@]2([C@H](C1(C)C)CC[C@@]1([C@@H]2CC=C2[C@@]1(C)CC[C@@]1([C@H]2[C@@H](C)[C@H](C)CC1)C)C)C	92,824	C_32_H_52_O_2_	
3	Stigmasterol glucoside	CC[C@@H](C(C)C)/C = C/[C@H]([C@H]1CC[C@@H]2[C@]1(C)CC[C@H]1[C@H]2CC=C2[C@]1(C)CC[C@@H](C2)O[C@@H]1O[C@H](CO)[C@H]([C@@H]([C@H]1O)O)O)C	6,602,508	C_35_H_58_O_6_	
4	Stigmasterol	CC[C@@H](C(C)C)/C = C/[C@H]([C@H]1CC[C@@H]2[C@]1(C)CC[C@H]1[C@H]2CC=C2[C@]1(C)CC[C@@H](C2)O)C	5,280,794	C_29_H_48_O	

### Phytochemical classification and hierarchical clustering

**Fig. 1:**
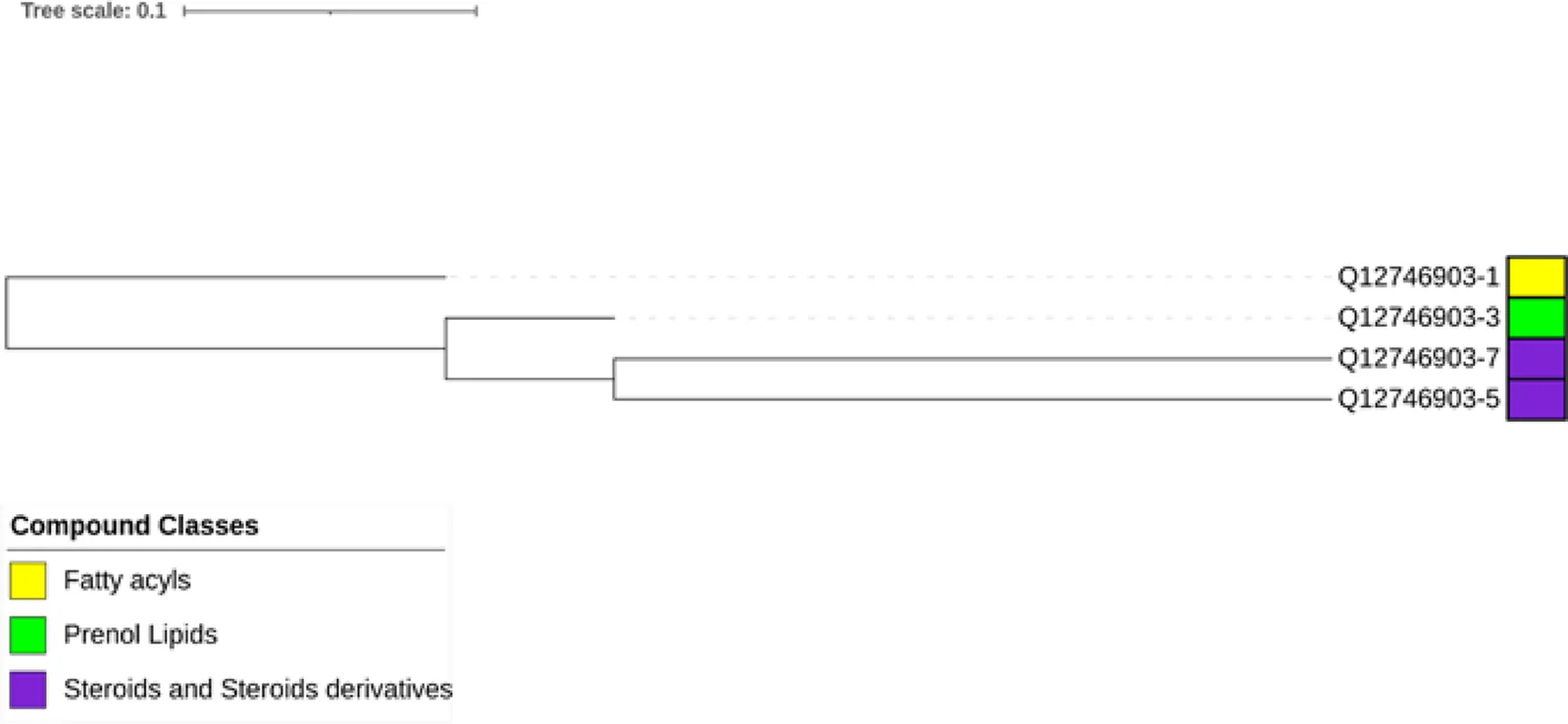
Chemical classifications of the compounds

The retrieved four phytochemicals from the database were assigned to analysis for their chemical classes. Automated chemical classification revealed that all the phytochemicals classified under the superclass lipid-like molecules. At the class level they show distinct three classes, where the compound 1 as tetracosanoic acid belongs to fatty acyls, compound 2 alpha-amyrenyl acetate classified in class prenols lipids and compound stigmasterol glucoside and stigmasterol belongs steroid and steroidal derivatives. Further subclass level classification reveals that compounds belong to fatty acids conjugates, triterpenoids, and stigmasterol derivatives. Revealing structural variations with lipid like molecules and chemicals classification of compounds shown in (Table S1), compounds hierarchical clustering revealed a structured organization of compounds based on their structural similarity (Fig. 1). Compounds with high chemical similarity clustered together indicating short branch lengths. Closely related steroidal compounds formed tight subcluster whereas, fatty acyls and prenol lipids position on more distant branches suggesting greater structural divergence.

### Drug likeness analysis

Drug-likeness evaluation of compounds based on their key physiochemical parameters including MW, HBD, HBA, log P, and TPSA. MW of the compound’s ranges from ~ 369 to ~ 575 gm/mol with stigmasterol glucosides exhibiting the highest molecular weight and tetracosanoic acid as the lowest. The number of HBD varied between 0 and 4, while HBA remains with acceptable range, compound shows variable log p values with tetracosanoic acid showing log p value > 10. tetracosanoic acid showing negative drug-likeness score except all three compounds exhibiting positive drug-likeness scores was showed in (Table 2).

**Table 2:** Drug-Likeness evaluation of the compounds

Phytochemicals	MW	HBD	HBA	MOL Log *p*	TPSA	Drug likeness score
Tetracosanoic acid	368.64	1	2	10.68	368.64	-0.54
alpha-Amyrenyl acetate	468.75	0	2	8.29	468.75	0.57
Stigmasterol glucoside	574.83	4	6	5.60	574.83	0.33
Stigmasterol	412.69	1	1	7.74	412.69	0.62

### ADME results

The ADME analysis results evaluate the pharmacokinetic properties of the four selected compounds. The results suggesting all the compounds are violating one Lipinsky rule, bioavailability estimates that tetracosanoic acid has with bioavailability (0.85), while the other three compounds with moderate predicted bioavailability (0.55). Whereas, tetracosanoic with a long chain fatty acid, with highly rotatability, while steroidal compounds need formulation to improve solubility or delivery, compounds with higher bioavailability. The four phytocompounds strongly differ in their GI absorption level, BBB permeability, and drug-likeness. stigmasterol, being a highly lipophilic steroid, generally with moderate GI absorption and can cross the BBB with slightly violating Lipinski’s rule (Table 3).

**Table 3:** ADME analysis of four phytocompounds.

Compounds	GI- Absorption	BBB	*P*-gp Substrate	CYP1A2	CYP2C9	CYP2D6	CYP3A4	Lipinski’s rule	Bio- availability
Tetracosanoic acid	Low	No	No	Yes	No	No	No	1	0.85
alpha-Amyneryl acetate	Low	No	No	No	No	No	No	1	0.55
Stigmasterol glucoside	High	No	Yes	No	No	No	No	1	0.55
Stigmasterol	Low	No	No	No	Yes	No	No	1	0.55

**Table 4:** Toxicity analysis of four phytocompounds

Compounds	Hepato- Toxicity	Nephro- Toxicity	Mutagenicity	reproductive toxicity	Immuno- Toxicity	Cyto- Toxicity
Tetracosanoic acid	Inactive	Inactive	Inactive	Inactive	Inactive	Inactive
alpha-Amyneryl acetate	Inactive	Inactive	Inactive	Active	Active	Inactive
Stigmasterol glucoside	Inactive	Active	Inactive	Inactive	Active	Inactive
Stigmasterol	Inactive	Inactive	Inactive	Inactive	Active	Inactive

Toxicity prediction of the four bioactive phytocompounds and safety profile was assessed before biological validation through a comprehensive in-silico toxicology assessment. ProTox-II toxicological analysis revealed variability in the toxicity profiles of the selected compounds. Toxicity assed through distinct toxic factors hepatotoxicity, nephrotoxicity, mutagenicity, reproductive toxicity, immunotoxicity, and cytotoxicity (Table 4). Organ specific toxicity, including hepatotoxicity, neurotoxicity, cardiotoxicity, respiratory toxicity, and with probability values ranging from moderate to high. All compounds showed non-toxic effects against all the factors except reproductive toxicity and immunotoxicity. Except tetracosanoic acid all other three compounds exhibited immunotoxicity, indicating potential systematics safety concerns, whereas alpha-amyrenyl-acetate predicted to be activated against estrogen receptor alpha (ER) estrogen receptor ligand binding domain (ER-LBD) suggesting potential endocrine-disrupting activity associated with reproductive developmental toxicity.

#### Prediction of toxicity class and LD_50_ value

Compounds toxicity class prediction showed that two compounds tetracosanoic acid and stigmasterol belongs to the class IV with LD_50_ values (900 mg/kg and 890 mg/kg, respectively) that suggesting that can be harmful if swallowed as per chemical toxicity class prediction; Whereas alpha-amyrenyl acetate predicted to be in class V with LD_50_ = 3460 mg/kg suggesting maybe or may not be harmful if swallowed; Compound stigmasterol glucoside refers to class VI, LD_50_ = 8000 mg/kg suggesting non toxicity (Table 5).

**Table 5:** Acute toxicity prediction with toxicity class and LD50 values

Sl No.	Compounds	Predicted LD50	Predicted Toxicity Class
1	Tetracosanoic acid	900 mg/kg	4
2	alpha-Amyneryl acetate	3460 mg/kg	5
3	Stigmasterol glucoside	8000 mg/kg	6
4	Stigmasterol	890 mg/kg	4

### Compounds antibacterial prediction

Four phytochemicals were investigated using the antiBac-Pred tool to predict potential antibacterial activity against 353 bacterial strains. This tool provides the names of the predicted susceptible bacterial strains along with confidence scores for each compound-bacteria interaction. A confidence score (Pa – Pi) greater than zero was considered as indicative of potential activity. The compound tetracosanoic acid resulted to be activated against *Yersinia*,* Shigella*,* Salmonella*,* Klebsiella*,* E.coli* and *Enterobacter* strains with confidence score ranging from 0.0078 to 0.524. Alpha-amyrenyl acetate shows inhibition against *Lactobacillus plantarum*,* Staphylococcus lugdunensis*,* Bacteroides stercoris*,* Clostridium ramosum* 0.541 to 0.3759.Whereas, stigmasterol glucoside predicted to be inhibit resistant strain of RESISTANT *Klebsiella oxytoca* and, RESISTANT *Providencia rettgeri*, RESISTANT *Citrobacter koseri*, RESISTANT *Acinetobacter pittii*,* Clostridium ramosum* with confidence score ranging from 0.29 to 0.5397. Stigmasterol compound showed inhibition against *Lactobacillus plantarum*,* Porphyromonas gingivalis*,* Kocuria rhizophila*,* Micrococcus luteu* confidence score ranging from 0.288 to 0.223. Enterobacterial infections are commonly associate with Gram-negative bacterial strains such as *Enterobacter spp.*,* Klebsiella*,* E. coli*,* Shigella*, based on prediction score. Two compounds tetracosanoic acid and stigmasterol glucoside showing inhibition against these strains, also other two phytocompounds shows multiple strain inhibition activity (Table 6).

**Table 6:** Antibacterial prediction of compounds with confidence score and strains

Phytocompounds	Strain	Confidence Score
Tetracosanoic acid	*Yersinia pseudotuberculosis*	0.0637
	*Yersinia pestis*	0.4192
	*Shigella sp.*	0.1298
	*Shigella dysenteriae*	0.0994
	*Salmonella enteritidis*	0.5247
	*Salmonella enterica* subsp. Enterica	0.1984
	RESISTANT *Yersinia enterocolitica*	0.0679
	RESISTANT *Salmonella typhi*	0.0451
	RESISTANT *Salmonella enterica subsp. Enterica*	0.0963
	RESISTANT *Providencia stuartii*	0.0463
	RESISTANT *Klebsiella oxytoca*	0.1646
	RESISTANT *Klebsiella*	0.0078
	RESISTANT *Escherichia coli* DH5α	0.0833
	RESISTANT *Enterobacter*	0.0921
	*Proteus inconstans*	0.1187
	*Proteus*	0.0259
	*Escherichia coli* DH5α	0.0187
alpha-Amyrenyl acetate	*Lactobacillus plantarum*	0.541
	*Staphylococcus lugdunensis*	0.5303
	*Bacteroides stercoris*	0.478
	*Bacillus subtilis* subsp. spizizenii ATCC 6633	0.3759
	*Clostridium ramosum*	0.3741
Stigmasterol glucoside	RESISTANT *Klebsiella oxytoca*	0.29
	RESISTANT *Providencia rettgeri*	0.1326
	RESISTANT *Citrobacter koseri*	0.0436
	RESISTANT *Acinetobacter pittii*	0.5534
	*Clostridium ramosum*	0.5397
Stigmasterol	*Lactobacillus plantarum*	0.2886
	*Porphyromonas gingivalis*	0.282
	*Kocuria rhizophila*	0.2541
	*Micrococcus luteus*	0.2237

### Compound and disease target prediction

**Fig. 2:**
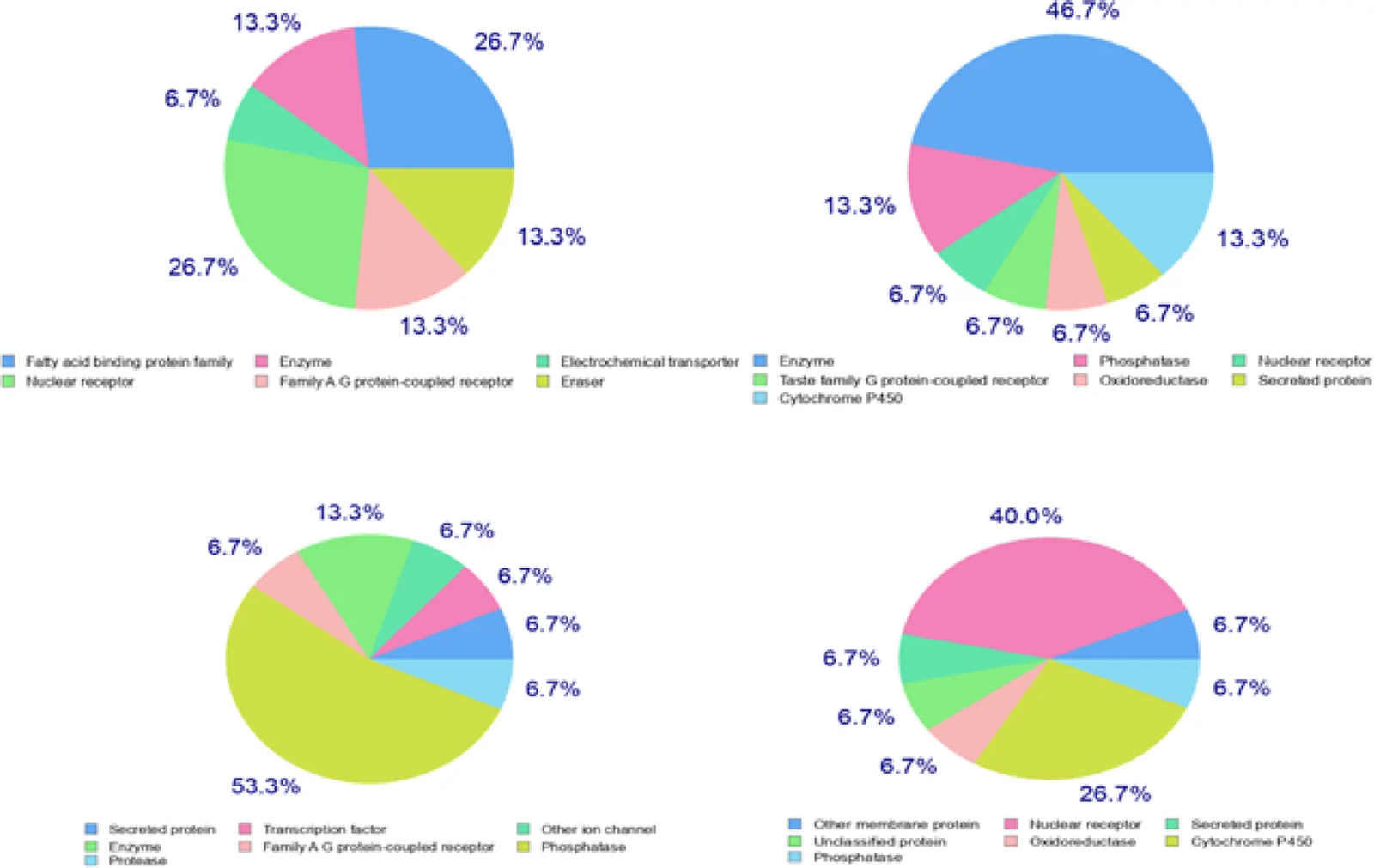
Swiss target prediction provided pie-chart representing the target classes of selected phytochemicals categorized in enzyme, family A G protein coupled receptor, phosphatase, nuclear receptor, secreted protein, and other functional protein families. Each target class’s probability-based forecast frequency is shown by the percentage distribution

Compound target prediction analysis showed that four phytochemicals revealed distinct but complementary pharmacological profiles. The SwissTargetPrediction analysis identified total 153 potential human targets for four selected phytocompounds of *H. javanica* at a probability threshold > 0. The predicted targets belonged to diverse functional classes including enzymes, kinase, G-protein-coupled receptors (GPCRs), nuclear receptors, proteases, and transporters. The first compound tetracosanoic acid predicted a total of 47- fatty acid binding proteins family, nuclear receptor, enzyme. Second compound alpha-amyrenyl acetate with total 41 targets that give a clear affinity for major targets classes and targets cytochrome p450, phosphatase, oxidoreductase, nuclear receptor, secreted protein HSD11B1, HSD11B2, PTGES, PTGS2 and CYP450. The third compound stigmasterol glucoside with total 23 protein targets, targeting phosphatases, enzyme, transcription factor, protease, family AG protein couple receptor CDC25 phosphatases and multiple HDAC isoforms. The stigmasterol compound predicted total 42 targets strongly associated with cholesterol transport and nuclear receptor signaling pathway with high probability involving NPC1L1, ESR11, and ESR2 (Fig. 2). Successfully unique 153 phytochemicals targets identification suggests the distribution of targets suggests potential multi-target pharmacological activity of the compounds that shows phytochemicals operate through multitarget mechanism. These total 153 targets of bioactive compounds used for further analysis. The potential targets for “Enterobacterial Infection” retrieve from the two databases (Fig. S1), including Genecards and CTD, predicting 28 potential targets from Genecards and total 14,080 targets from CTD.

### Intersectional analysis of compound-predicted targets and disease associated targets

**Fig. 3:**
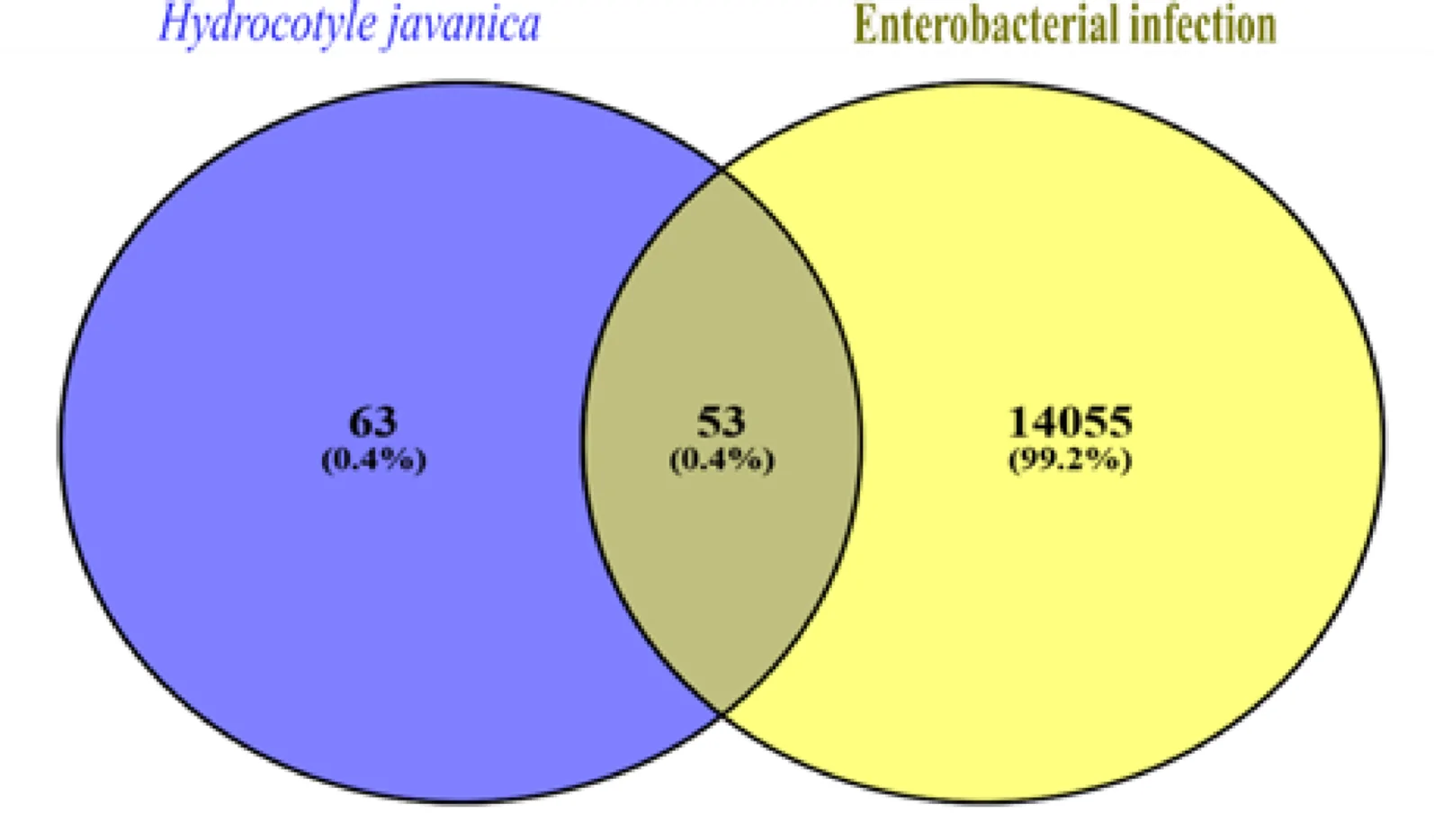
Venn diagram representing interaction beween selected compounds predicted targets and disease related targets

**Fig. 4:**
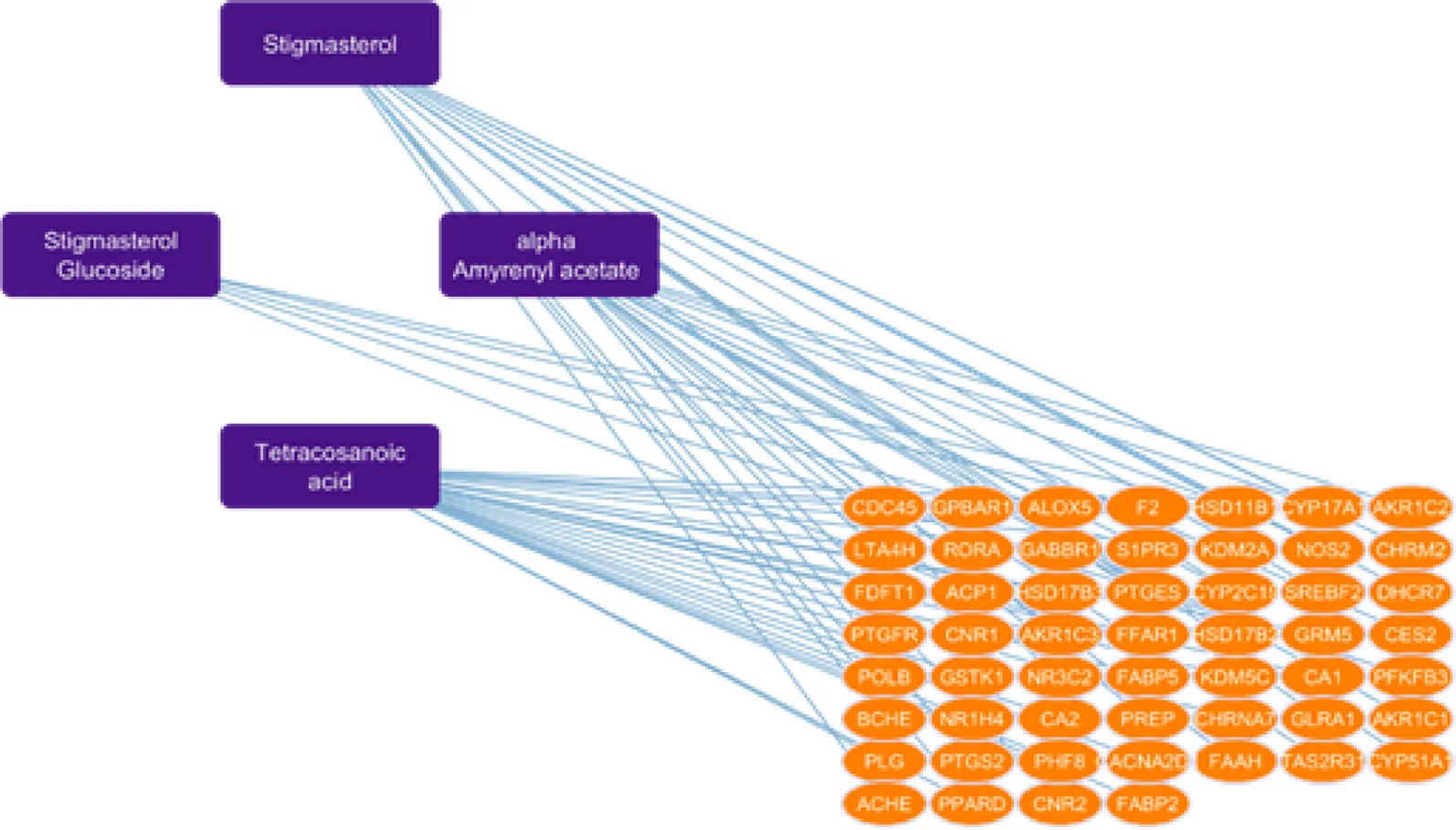
Compounds-target interaction network illustrating predicted bioactive compounds in purple color interaction with overlapping targets showing in orange color indicating their potential interactions

After merging two datasets total 14,108 potential targets for “Enterobacterial infection” were obtained. The common intersecting targets between disease and phytocompounds datasets 14,108, and 153, respectively using Venn diagram tool, resulted total 53 potential proteins targets (Fig. 3 and 4) representing core therapeutic targets, and these 53 gene targets were further used for GO and KEGG enrichment analysis and network construction.

### PPI analysis

**Fig. 5:**
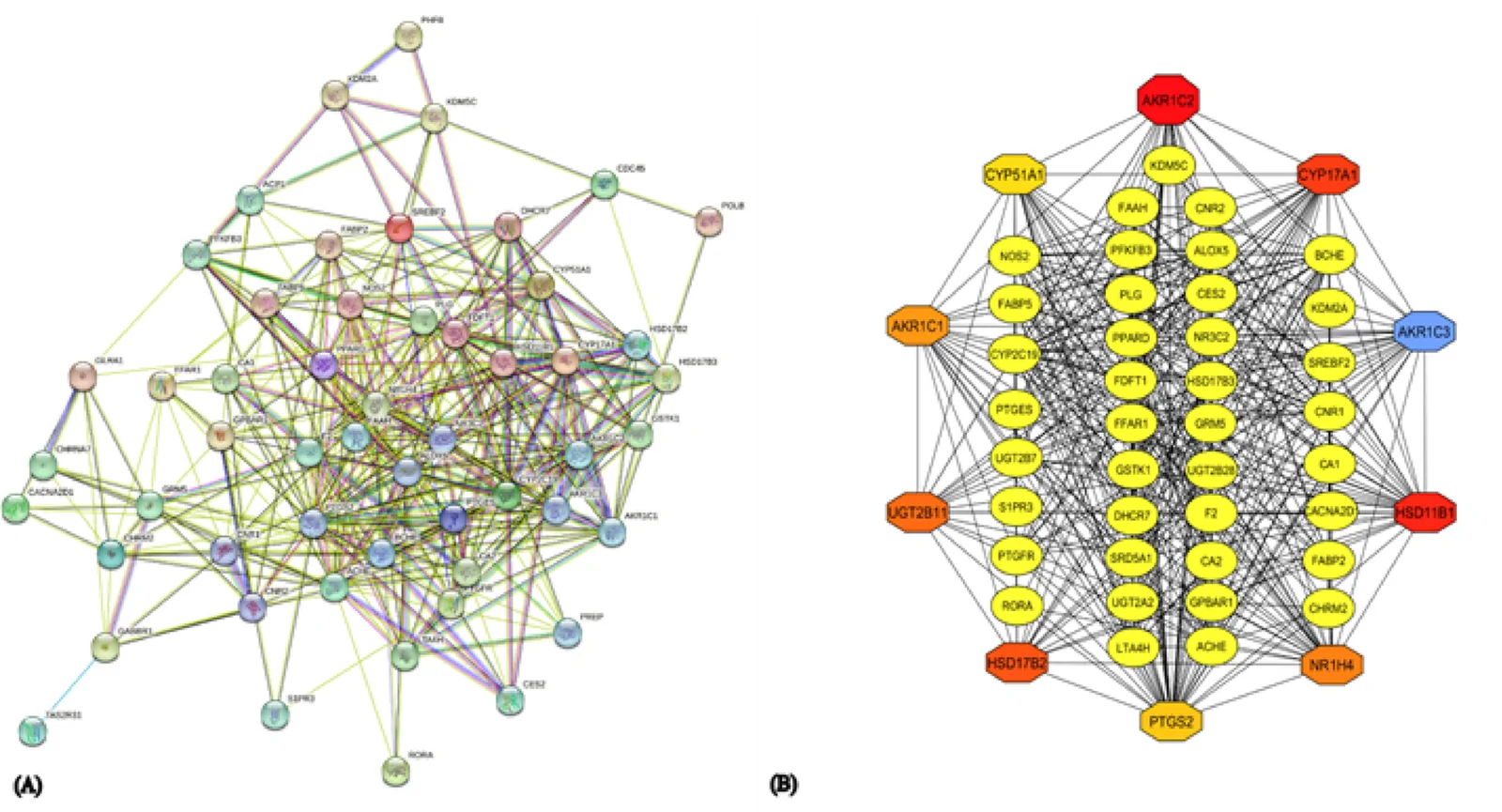
PPI network of 53 common targets. (A)PPI network constructed using STRING database, illustrating functional interactions among common targets. (B) Common targets constructed PPI network visualized in Cytoscape.

**Fig. 6:**
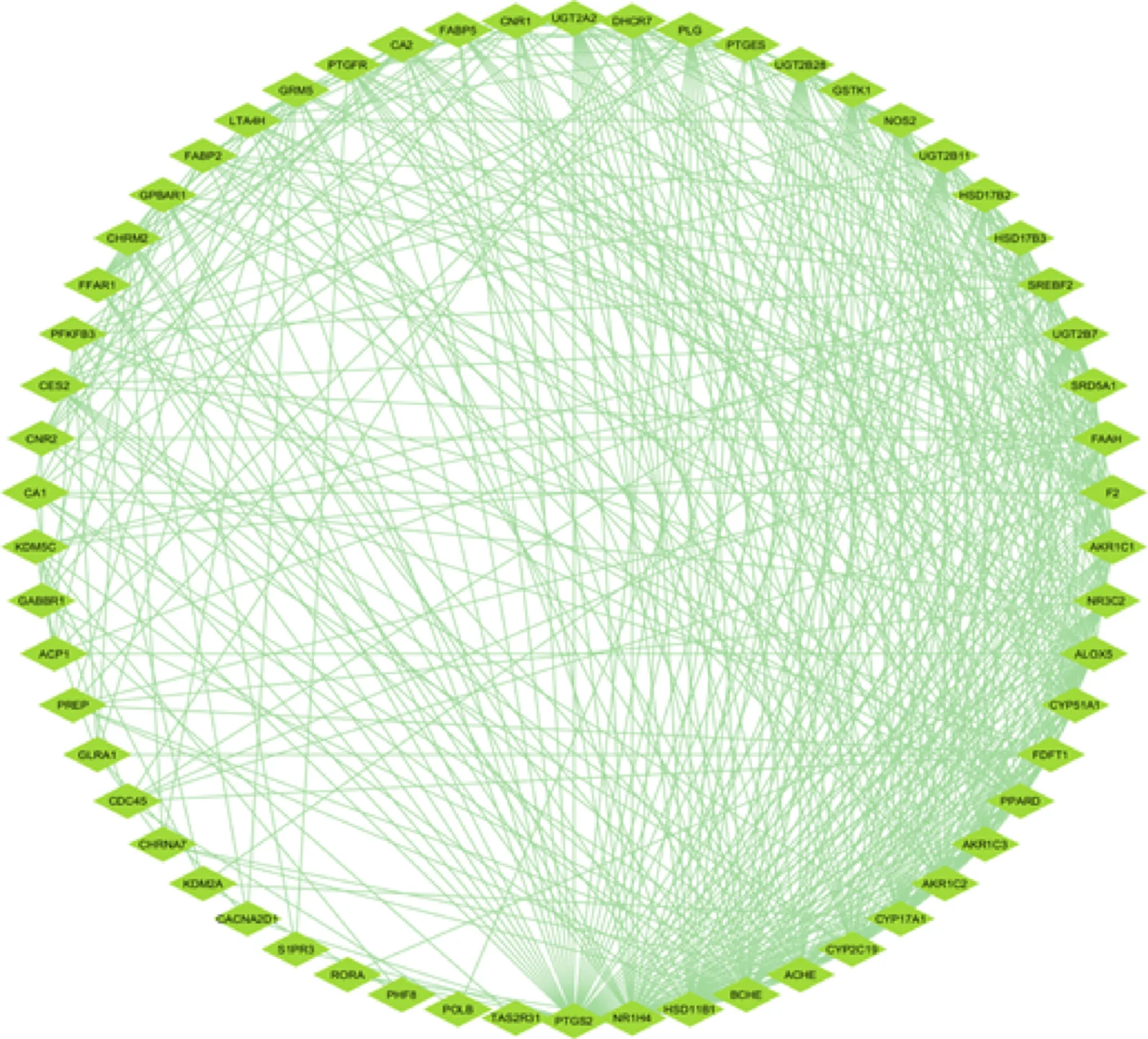
Circular layout representation of 53 potential targets interaction network constructed and visualized in Cytoscape where nodes represent targets and edges indicating interactions

The 53 common targets were analyzed for PPI network using String database and further network visualized by importing network into Cytoscape software. The PPI interaction network shows 53 nodes which represent the individual target protein and 376 edges which shows interactions between them. Topological analysis was performed to characterize the overall network construction with an average node degree of 14.2, higher degree values are often used to define strong association and help to identify hub targets. The network represents a tendency toward clustering with an average local clustering coefficient value of 0.524, which indicates that the interaction within the network is non-random. The random network model was also calculated with the expected number of edges 105, and the observed number of edges 376, which is higher than the expected number, and showing the complexity and biological relevance of the interactions. The PPI enrichment* p*-value > 1.0e-16 suggests that proteins are biologically interconnected (Fig. 5and 6).

### Network construction

**Fig. 7:**
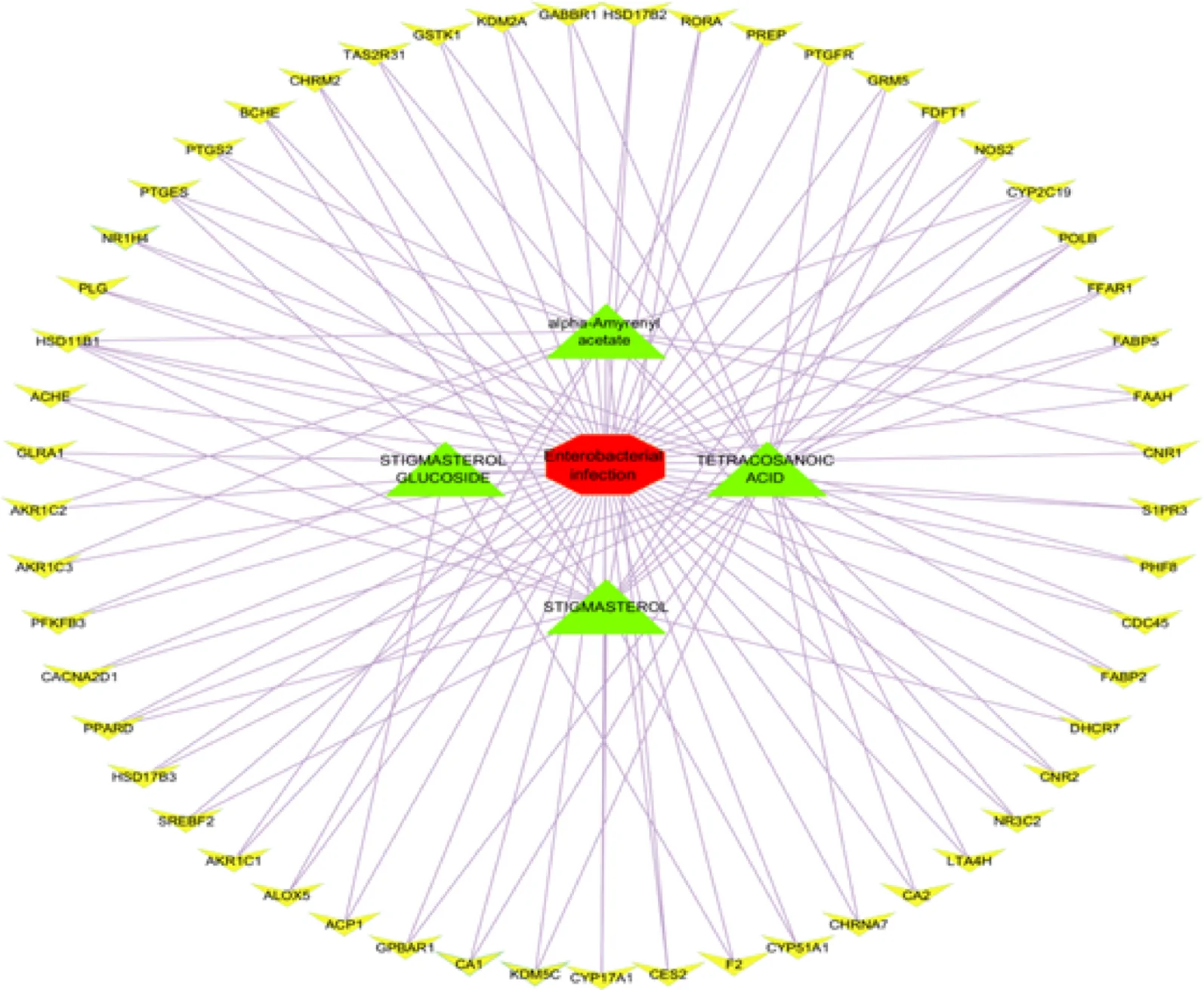
Compound-Targets-Disease interaction network, the nodes representing Enterobacterial infection in red color in the placed at center green color nodes representing retrieved phytocompounds in triangular shape and potential targets at the periphery in yellow color

A Compound-Target-Disease(C-T-D) network (Fig. 7) was created using Cytoscape to evaluate the multi-targets mechanism of the selected phytochemicals. The nodes in the network represent infections, four selected phytochemicals, and potential therapeutic targets, whereas the edges represent the interaction between these nodes and their relationships.

#### Hub-target analysis

**Fig. 8:**
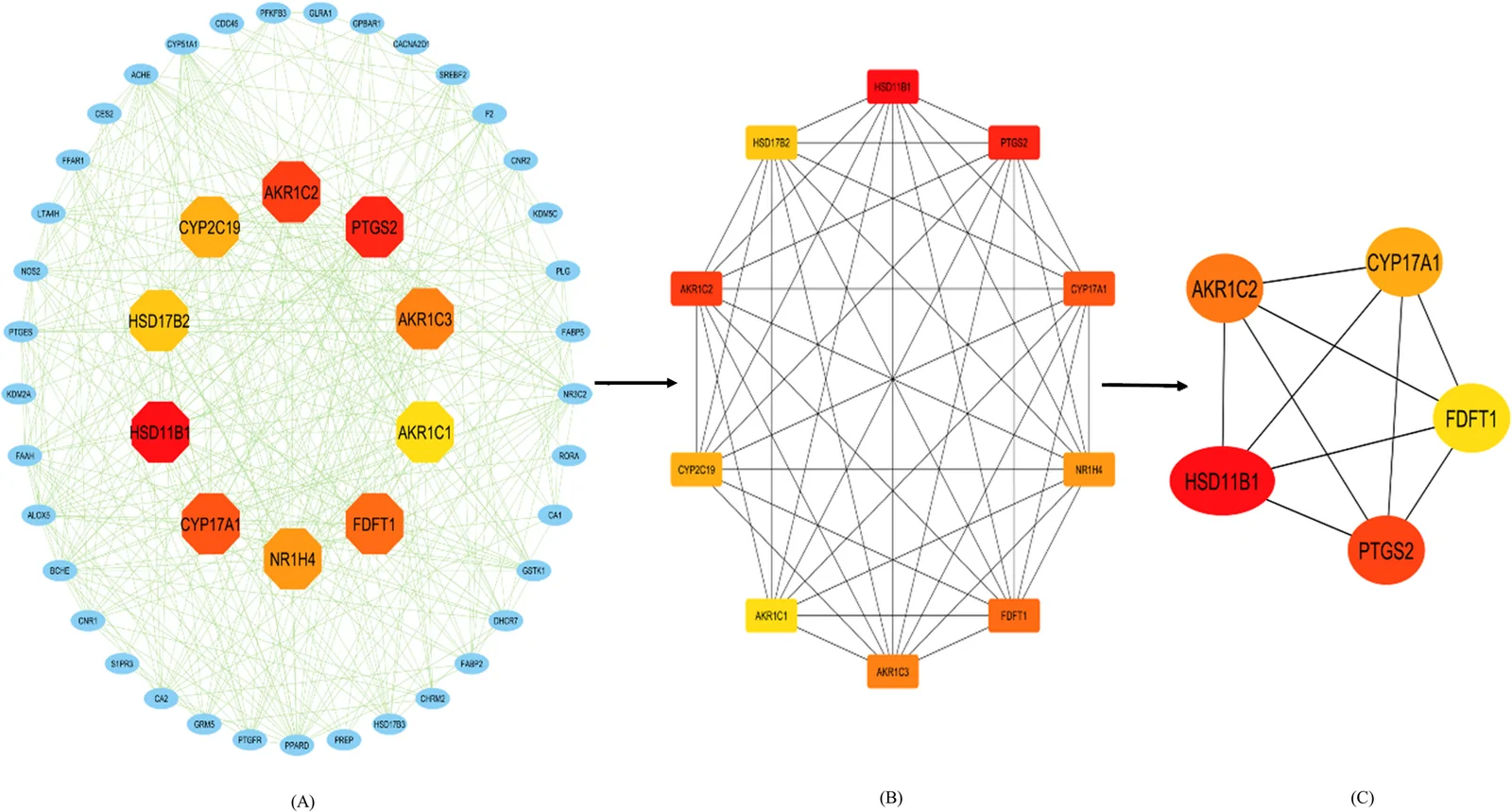
Hub-targets analysis (**A**) Potential targets interaction network with hub-targets, (**B**) top 10 hub-targets, (**C**) Top 5 hub-targets

The potential top key regulatory targets were identified in Cytoscape using CytoHubba plugin. The Hub genes were ranked based on MCC value. The top 5Hub targets [HSD11B1, PTGS2, AKR1C2, CYP17A1, FDFT] identified based on their interactions score (Fig. 8).

### Enrichment analysis

### GO functional annotation of overlapping targets

**Fig. 9:**
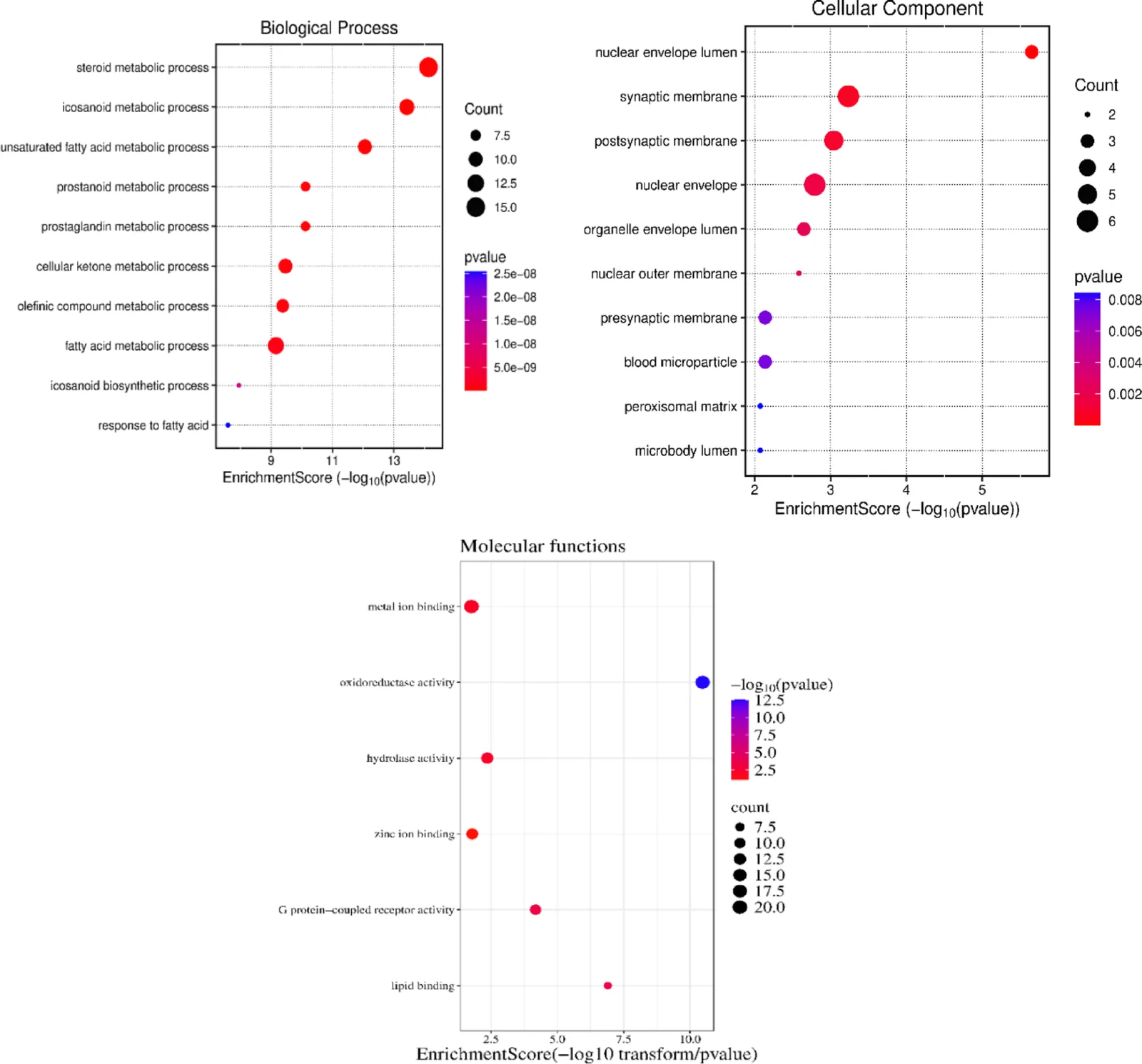
GO enrichment analysis, bubble plots showing top enriched GO terms categorized in biological process, cellular components, molecular functions based on enrichment scores

GO enrichment was performed using DAVID annotation tool, where 53 common targets were uploaded using the official gene-symbols and analysed gene ontology under BP, CC, and MF categories total 77BP, 20CC, 56MF obtained initially then filtered out using the threshold set to ≤ 0.05 and arrange to largest to smallest total 59 biological function, 14 cellular component and 44 molecular function terms obtained. The enriched BP showed host defence and metabolic adaptation mechanism including G protein-coupled receptor signalling pathway lipid metabolic process, inflammatory response, cholesterol metabolism, response to oxidative stress and steroid metabolic processes. CC indicating strong localisation of membrane associated and signalling domains, including plasma membrane, endoplasmic reticulum, extracellular exosomes, postsynaptic membrane, and nuclear envelope lumen. MF enrichments highlight the oxidoreductase functions, dehydrogenase activity, monooxygenase activity, and receptor binding indicating that the compounds can modulate oxidative balance, lipid metabolism, and hormone linked immune pathways (Fig. 9) and GO analysis bar plot present in (Fig. S3).

### KEGG pathway enrichment analysis

**Fig. 10:**
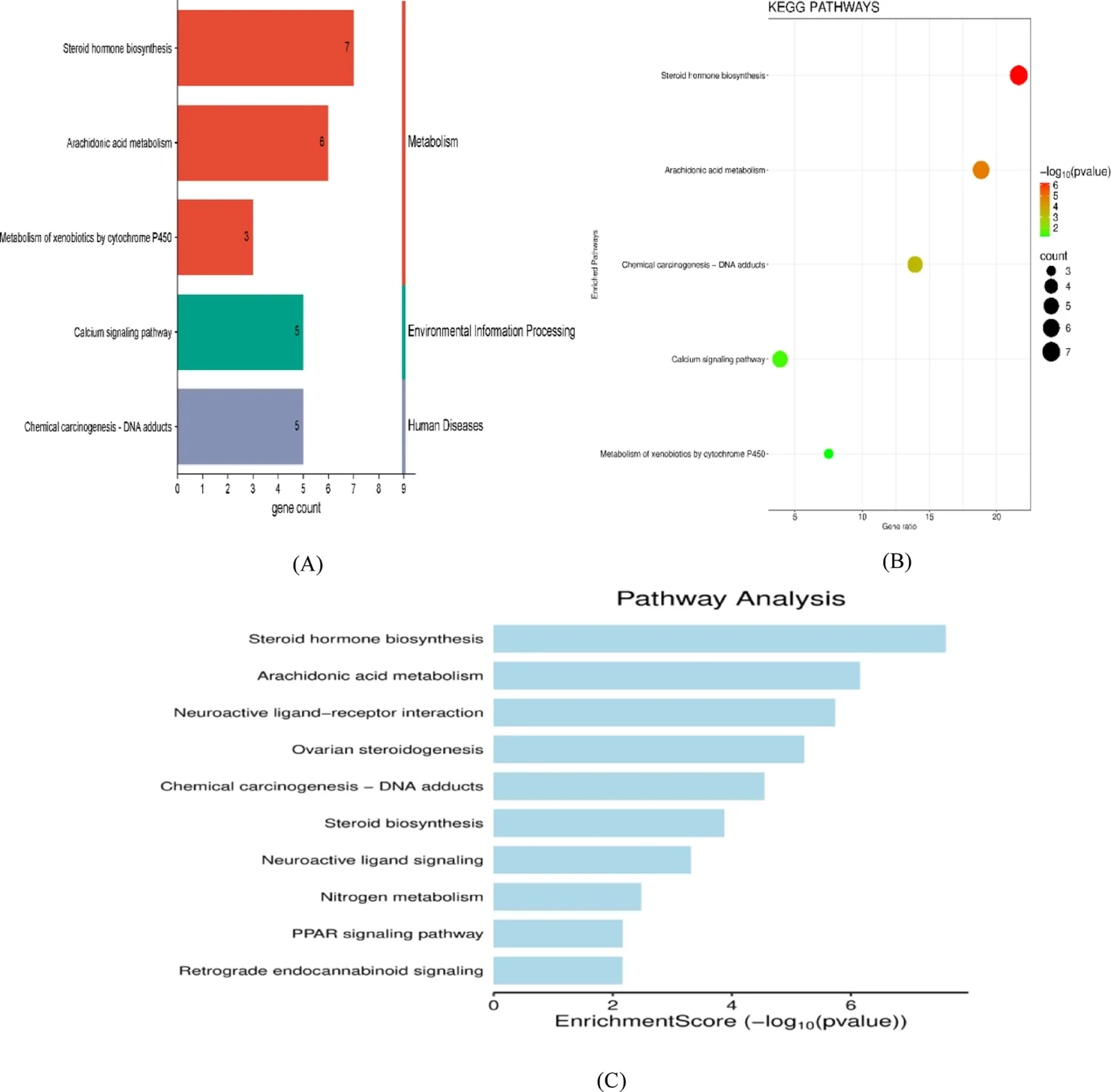
KEGG pathway enrichment analysis, (**A**) A bar plot showing the biologically relevant (**B**)Dot plot showing the enrichment of key biologically relevant pathways where x axis gene count and y axis with pathways name (**C**) Bar chart showing top enriched pathways identified

**Fig. 11:**
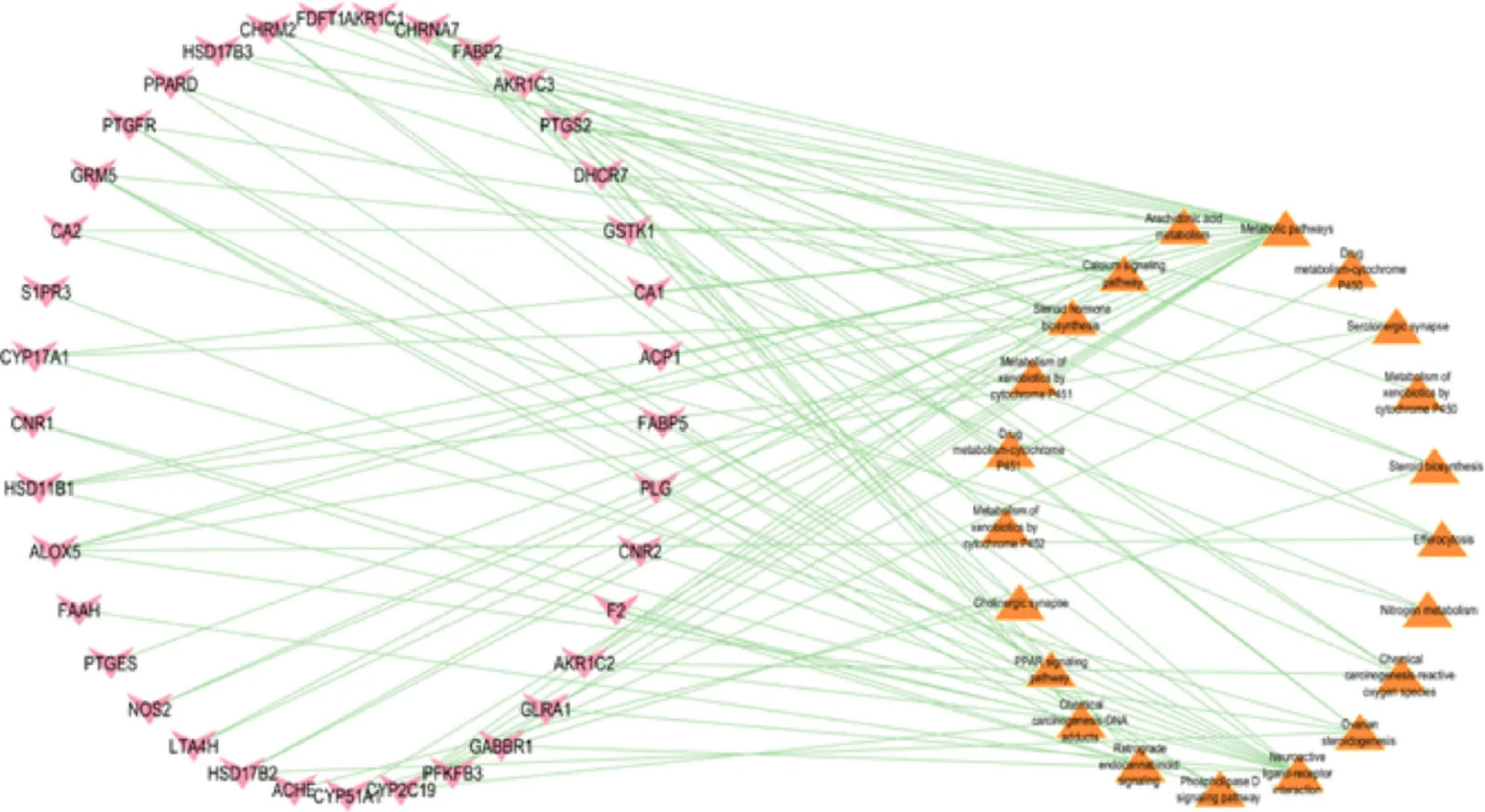
Target-Pathway interaction network, nodes representing pathways in orange colour and potential target genes in magenta colour.

The 53 common targets of *H. javanica* derived bioactive compounds and enterobacterial-infection related genes were subjected to pathway enrichment analysis revealed total 228 KEGG pathways from enrichment analysis summary. Out of these, 13 significant enriched pathways were meeting the threshold of *p* ≤ 0 steroid hormone biosynthesis, arachidonic acid metabolism, chemical carcinogenesis - DNA adducts, calcium signalling pathway, metabolism of xenobiotics by cytochrome P450 showed significant enrichment showed in enrichment plots (Fig. 10). Also, pathway-target interaction analysis uncovered several signaling pathways related to host immune response and infection mechanisms including phospholipase D signaling pathway, calcium signaling pathway, chemical carcinogenesis-reactive oxygen species, PI3K-Akt signaling pathway, MAPK signaling pathway, pathogenic *E. coli* infection, TNF signaling pathway (Fig. 15). These relevant pathways targeting key hub targets genes (Fig. 11) identified in network analysis and recognized for host-pathogen interactions and chosen for molecular docking analysis. pathways with their corresponding gene counts and classified in major classes.

### Molecular docking

**Fig. 12:**
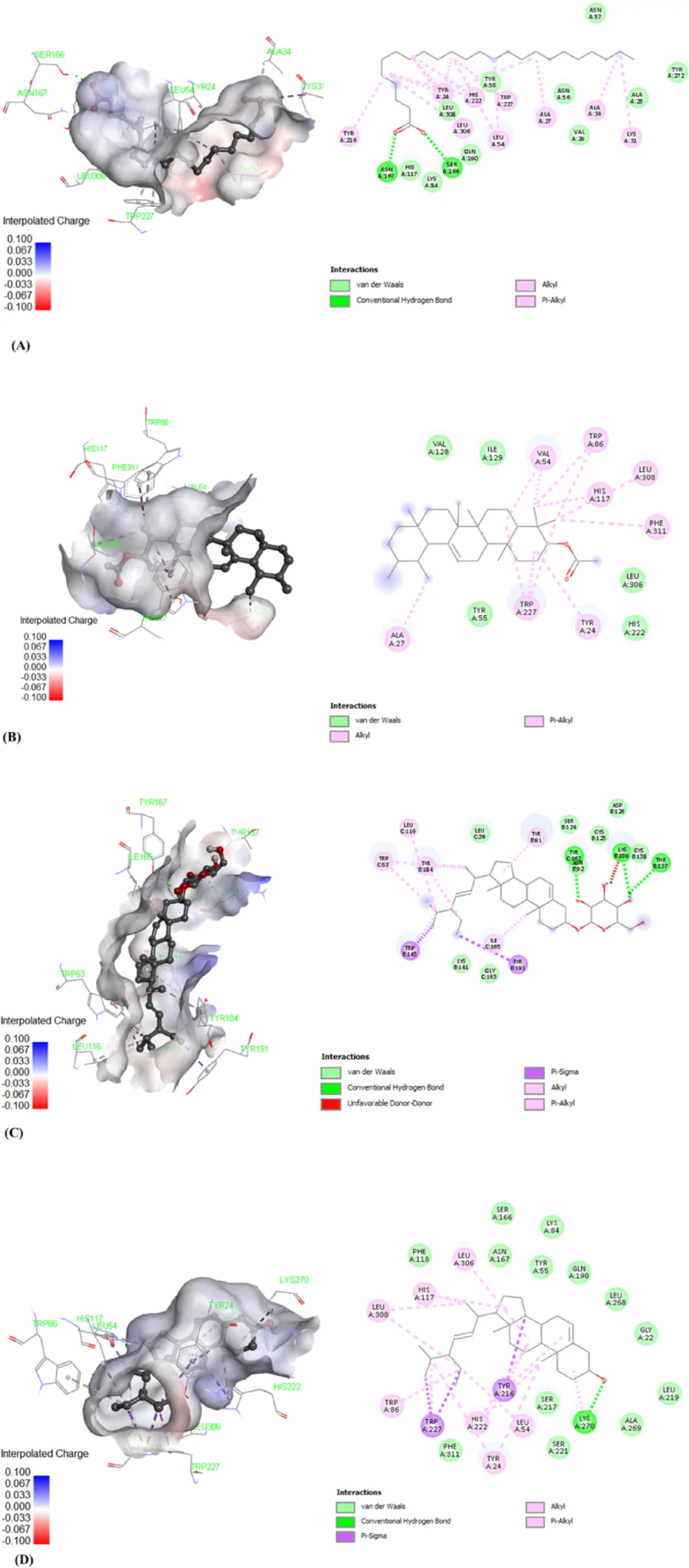
Molecular interactions and docking poses of selected phytocompounds as ligands in different sites of the protein receptors, where showing surface representation of protein with ligand binding at the active site, Three-dimensional view of protein-ligand interactions highlighting key interacting lines, 2D interaction diagram of ligand-receptor with relevant residues of (**A**) 3C3U and tetracosanoic acid, (**B**) 2HDJ and alpha amyrenyl acetate (**C**) 5OUG and stigmasterol glucoside, (**D**) 3C3U and stigmasterol

Molecular docking was performed between four phytocompounds of *H. javanica* as ligands and 11 target proteins as receptors in PyRx software. The protein treated as rigid and ligand remains flexible so it can twist or modify according to its bond and interactions. The software shows multiple binding scores ranked accordingly, to their binding affinity. The top-ranked score of the conformation demonstrated the lowest or most negative binding energy, indicating the most favourable interaction with the receptor. The docking score for each ligand and protein resulted in (Table 7), the docking energies ranging from (−3.4 to −10.7 kcal/mol) indicate the notable binding potential for all targets. The Vina wizard engine predicts how ligand binds to the receptor’s active site. Across all four compounds, stigmasterol displayed strongest and most favourable binding affinity with docking scores exceeding − 9.0 kcal/mol, 3C3U (-10.7 kcal/mol), 5OUG (-10.1 kcal/mol), stigmasterol glucoside exhibiting binding affinities with scores ranging from (−6.7 to −10 kcal/mol) showing high affinity with 5OUG, 5ZK8, 3C3U, 19GS; whereas alpha-amyrenyl acetate shows moderate binding affinities range from (-7.2 kcal/mol to -9.9 kcal/mol) showing highest affinity with 2HDJ, in contrast ligand 1 as tetracosanoic acid shows consistently demonstrated the weakest interaction with docking scores ranging from (−3.4 to −6.9 kcal/mol) showing lowest affinity with 3HR4 and highest affinity with 3C3U in (Fig. 12).

**Table 7:** Molecular docking scores with binding affinity 3D structures of receptors

Gene name	PDB ID	3D structure	Tetracosanoic acid Binding affinity (kcal/mol)	alpha-Amyrenyl acetate Binding affinity (kcal/mol)	Stigmasterol glucoside Binding affinity (kcal/mol)	Stigmasterol Binding affinity (kcal/mol)
PTGS2	5IKT		4.1	−8.2	-8.6	7.4
F2	6O39		−4.8	−9.5	-8.7	−8.8
CACNA2D1	19GS		−5.3	−9.3	−9.1	−8.9
AKR1C3	1ZQ5		−5.3	−7.4	−8.2	−8
ACP1	7KH8		−5.3	−7.2	−6.7	−7.1
AKR1C2	2HDJ		−6.7	−9.9	−8.6	−9.4
AKR1C1	3C3U		−6.9	−8.3	−9.1	−10.7
NOS2	3HR4		−3.4	−8.4	−7.7	−8.6
GRM5	6N4X		−4.3	−8.6	−8.8	−8
CHRNA7	5OUG		−6.7	−9.7	−10	−10.1
CHRM2	5ZK8		−5.7	−9.6	−9.5	−8.9

### Molecular dynamics simulation

**Fig. 13:**
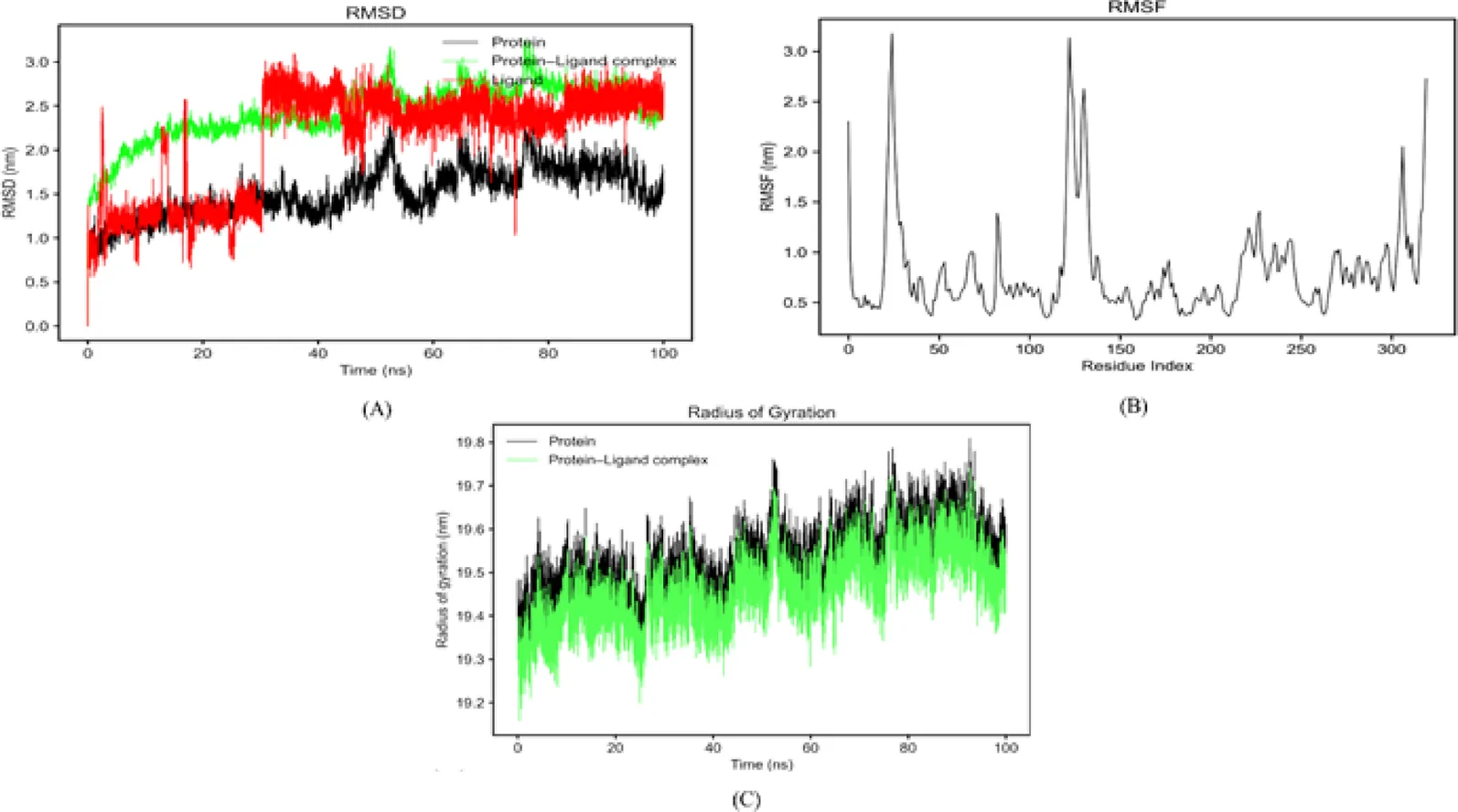
MD simulation of selected protein-ligand complex (3C3U-Stigmasterol). (**A**) RMSD plot of protein-ligand complex against time (ns.) through simulation production; (**B**) RMSF residues level flexibility of protein; (**C**) Rg plot showing compactness and conformational stability

MD simulations that has been widely used for various studies to validate protein ligand stability in drug discovery (Mandal et al. 2021). Performing MD simulation, the stability and the flexibility of the protein-ligand complex evaluated under physiological conditions over time. The top ranked protein/receptor 3C3U and the highest binding affinity and ligand stigmasterol molecule complex obtain from the docking experiment. MD simulation carried for 100 ns. to understand the behavioural pattern of protein-ligand complex under simulated physiological conditions. These trajectories calculated including:

RMSD the structural stability of protein, ligand, and protein–ligand complex evaluated during the 100 ns molecular dynamics simulation using RMSD analysis (Fig. 13A). 3C3U protein the protein RMSD showing in black stays around ~ 1.2–1.8 nm, initially increased during the early phase of the simulation reflecting structural relaxation, and eventually stabilized after approximately 20–25 ns, this stabilization indicates that the protein attained a stable conformation during the production run. The protein–ligand complex exhibited a slightly higher RMSD compared to the 3C3U protein, protein-ligand complex in green rise initially then plateaus after approximately 30 ns ~ 2.2–2.6 nm with values stabilizing. The consistent RMSD profile without abrupt deviations suggests that ligand binding did not induce major conformational destabilization of the protein and that the complex remained structurally stable throughout the simulation. The ligand RMSD in red colour showed moderate fluctuations during the initial phase of the simulation, get equilibrated after approximately 25–30 ns, indicating that the ligand adjusted within the binding pocket and maintained a stable binding orientation over the remaining simulation time. RMSF analysis performed to evaluate residue level flexibility of the protein during 100ns simulation. The RMSF plot (Fig. 13B) revealed x axis representing residue index and whereas y-axis represent RMSF (nm), major high fluctuation regions with prominent peaks showed at residues with RMSF value ~ 2.5–3.0 nm which is present at the N-terminal region. The loop region showed sharp spike with RMSF value ~ 3.0 nm at ~ 35–45 residues, and multiple high peaks visible residues at ~ 100–150 with RMSF value ~ 2.5-3.0, residues ~ 240–260 with moderate peaks at ~ 1.5–2.0 nm. At the C-terminal region shows ~ 2.5 nm RMSF value at ~ 270–290. At residues ~ 50–110 and residues 150–220 shows lower RMSF values below ~ 0.5-1.0. There is NO widespread high fluctuation across the protein, which means no unfolding or instability. The RMSF profile revealed that most residues exhibited relatively low fluctuations, indicating a stable protein backbone. Higher fluctuations were observed predominantly at the N-terminal region (residues ~ 1–15), selected loop regions (residues ~ 35–45 and ~ 115–135), and the C-terminal region (residues ~ 270–290) and reflecting the inherent flexibility of these segments. In contrast, residues corresponding to the core regions of the protein displayed lower RMSF values, suggesting structural rigidity and stabilization upon ligand binding. Overall, the RMSF results indicate that the protein maintains a stable conformation while allowing necessary flexibility in peripheral regions during the simulation. High RMSF values were mainly observed in terminal and loop regions, while core residues showed low fluctuations, indicating stable ligand binding and overall protein stability. Radius of gyration calculation suggest the compactness of the protein structure during simulation the Rg value of complex remain constant through the 100 ns simulation detailing the overall structure maintaining the gyration radius curve represents the compactness of the overall structure of the protein. Analysis of Rg was performed to understand the overall compactness of the protein structure during the simulation (Fig. 13.1 C). An increase in Rg values typically indicates that the protein structure becomes loos or unfolds, while stable Rg values suggest that the protein maintains its overall structural compactness. In the plot the Y- axis represent the ligand X-axis represent time(ns). The protein 3C3U and the complex Rg calculation suggest the compactness of the protein structure during simulation the Rg value of complex remain constant through the 100 ns simulation detailing the overall structure maintaining.

**Fig. 14:**
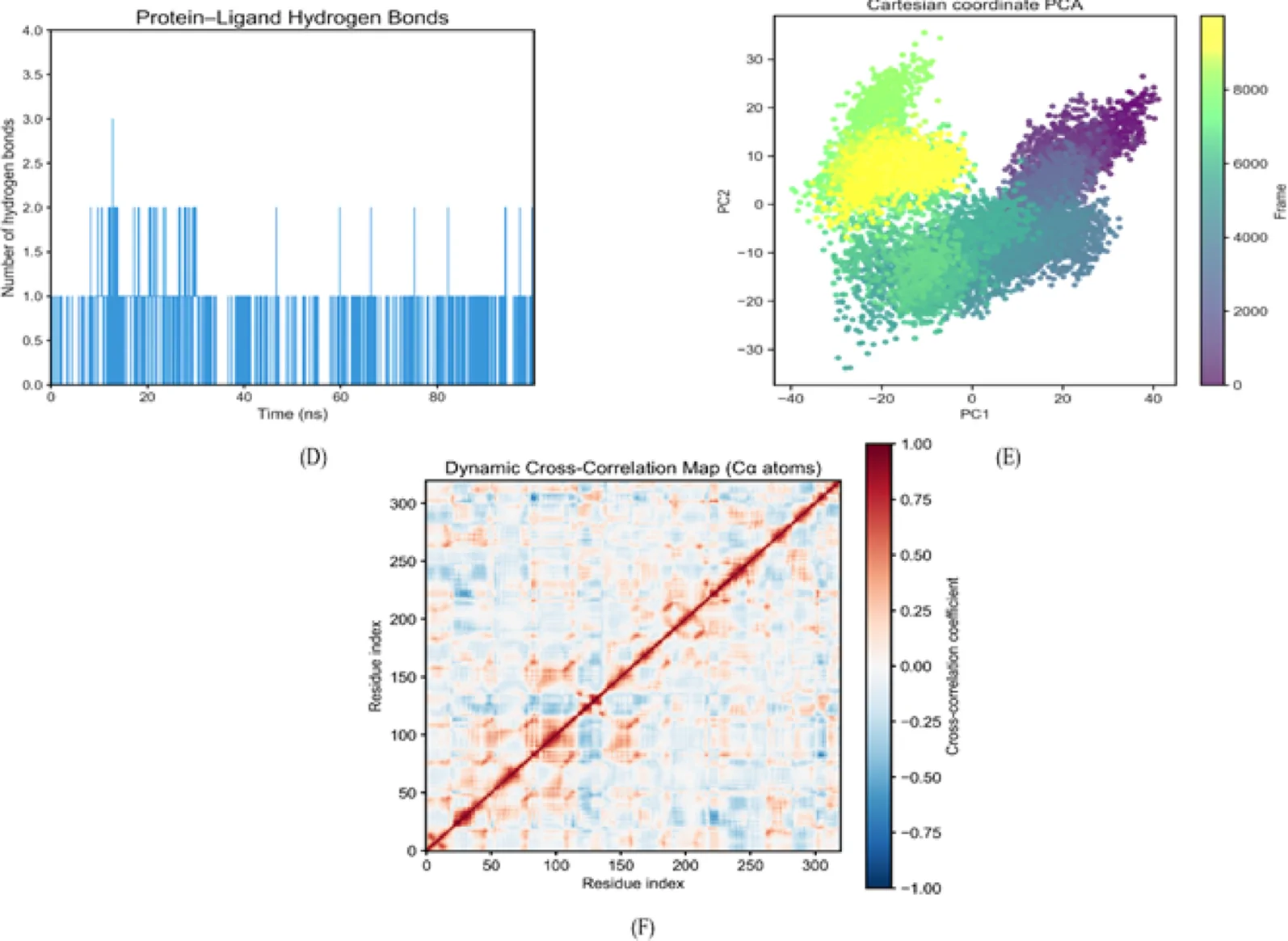
MD simulation of protein-ligand complex (3C3U-Stigmasterol), (**D**) Protein-ligand hydrogen bond analysis showing the number of hydrogen bonds formed during simulation production; (**E**) PCA representing conformational motions of complex; (**F**) plotting DCCM residue-residue motion correlation

Protein ligand H bond analysis carried out to evaluate interaction stability using simulation hydrogen bond analysis was performed to evaluate the stability of interactions between the protein and ligand during the 100 ns MD simulation (Fig. 14D). The protein–ligand complex maintained a consistent number of hydrogen bonds throughout the simulation, indicating stable binding of the ligand within the active site. The plot revealed X axis representing time (ns.) and Y-axis representing no. of hydrogen bonds the average number of hydrogen bond is 1 to 2 and occasional formation of number of hydrogens bond up to 3. The persistence of at least one hydrogen bond during most of the simulation suggests stable ligand binding within the active site. The observed fluctuations in hydrogen bond number reflect the dynamic nature of protein–ligand interactions rather than binding instability. Overall, the hydrogen bonding pattern supports a stable and favourable interaction between the protein and the ligand. PCA was carried out to investigate the essential collective motions of the protein during the 100 ns. MD simulation (Fig. 14). The projection of the trajectory onto the first two principal components (PC1 and PC2) revealed a compact and tightly clustered distribution. The restricted conformational space observed in the PCA plot indicates limited large-scale structural fluctuations and suggests that the protein–ligand complex remains dynamically stable throughout the simulation. The absence of widely scattered conformations further confirms that ligand binding effectively stabilizes the protein and constrains its collective motions. Overall PCA analysis demonstrates that the protein–ligand complex exhibits stable and coherent dynamics and restricted conformational space and enhanced stability of the protein-ligand complex during the simulation period. In DCCM During 100 ns. MDS based on C atoms analysed correlated motions among protein residues. The correlation among the protein residue was investigated by a cross-correlation matrix, that means how differently residues of the protein move with respect to each other during MD simulation. Fig. 14F explains the positive correlated red regions where the two residues move in the same direction, and the negatively correlated blue regions where two residues move in different direction and the white regions has no relation between movements, whereas the diagonal line represents every residue perfectly moves with itself. The plot shows both positively and negatively correlated motions among residues indicating coordinated and flexible movement of the protein. The DCCM plot revealed strong and positive correlation along the diagonal, which refers strong consistent residue wise movement. Several correlated movements suggesting coordinate movement among protein structural domains, also several anti-correlated or negatively correlated regions observe this suggesting protein behave a balanced, coordinate, and flexible movements upon ligand bind. Presence of positive correlated motions higher than the anti-correlated motions the residues within ligand binding regions showed coordinate motions indicate ligand bind stabilized internal residues communication without disrupting the protein dynamics.(Fig. 14)

## Discussion

**Fig. 15:**
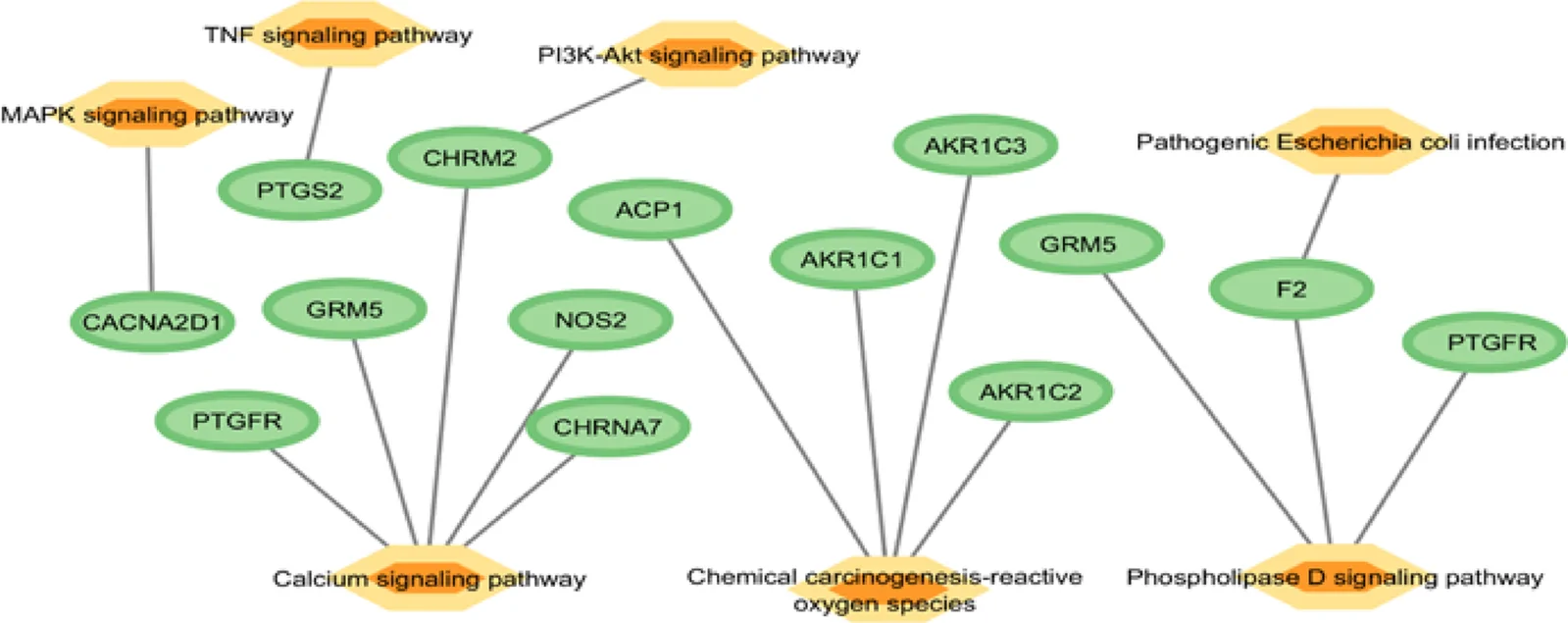
Potential pathways with Hub-targets.

Enterobacter species represent the sixth leading cause of nosocomial infections worldwide, often resulting in mortality rates as high as 80% in neonatal meningitis with (Salimiyan Rizi et al. 2020) the clinical challenge is severely compounded by multifactorial multidrug resistance (Anju et al. 2020).The study explored the therapeutic multitarget potential of *H. javanica* against enterobacterial infections using a systematic network pharmacology framework integrated with chemical classification, ADME–toxicity profiling, and PPI network analysis, molecular docking, and MD simulation. The findings demonstrated that key plant derived bioactive compounds viz. tetracosanoic acid, alpha-amyrnyl acetate, stigmasterol glucoside, and stigmasterol interact with multiple host-associated targets involved in immunity, inflammation, bacterial response, and membrane integrity. Supporting a host-modulatory, multi-target therapeutic mechanism with combined phytochemical approaches for improving therapeutic efficacy(Abiraamavalli and Namasivayam 2025). This in-silico network pharmacology approach with phytochemicals give a holistic view of multi-target drug action by shifting the focus from single-target cascades to coordinated responses across complex cellular networks (Berger and Iyengar 2009). Our study starts with chemical classification of the four selected phytocompounds resulting primarily belongs to the lipid derived classes fatty acyls, prenol lipids, steroid, and steroidal derivatives. The in-vitro study of *H. javanica* reported antimicrobial activity associated (Mandal et al. 2016) with alkaloids and phenols and terpenoids, where prenol lipids steroidal compounds are biosynthetically related to terpenoids. The steroidal compounds being triterpenoid derivatives shows reported antimicrobial activity in plants. Drug-likeness prediction of compounds showcase one compound exceeding the range of molecular weight indicating a possible limitation in oral bioavailability (Veber et al. 2002), one compound with high log P suggesting high lipophilic with poor solubility (Lipinski 2000), all four compound showing one Lipinski’s rule violation as popular druggable compounds with one rule violation still showing drug like properties (Ivanović et al. 2020), overall most compounds with evaluated acceptable physiochemical properties with limited rule violations. In computational drug discovery, ADME prediction is extensively used to assess the pharmacokinetic characteristics and drug-likeness of potential lead compounds (Singh et al. 2026). The ADME data highlights cytochrome P450 inhibition profiles of selected compounds indicating favourable metabolic safety with minimize the risk of drug-drug interaction (Steyn and Varma 2020), most of the compounds is not inhibiting CYP isoforms. P-glycoprotein substrate prediction only one compound was identified as P-gp substrate indicating efflux transport and reduce intracellular contraction(Varma et al. 2005)remaining compounds being non substrate suggest better absorption and distribution. Target prediction analysis revealed large number of diseases associated targets compared to compounds predicted targets showing total 53 common targets between them. These common targets may play crucial role in the therapeutic pathways against the disease, Fig. 4a showing the relation between compounds and with common targets. GO analysis suggest that compound’s predicted targets are significantly associated with key biological processes involving oxidative stress, hormone signalling, inflammatory response and show case therapeutic mechanism with multiple interlinked molecular function responses and cellular component showcasing plasma membrane, endoplasmic reticulum, nuclear receptors reflecting targets are widely distributed.(Fig. 15)

**Fig. 16:**
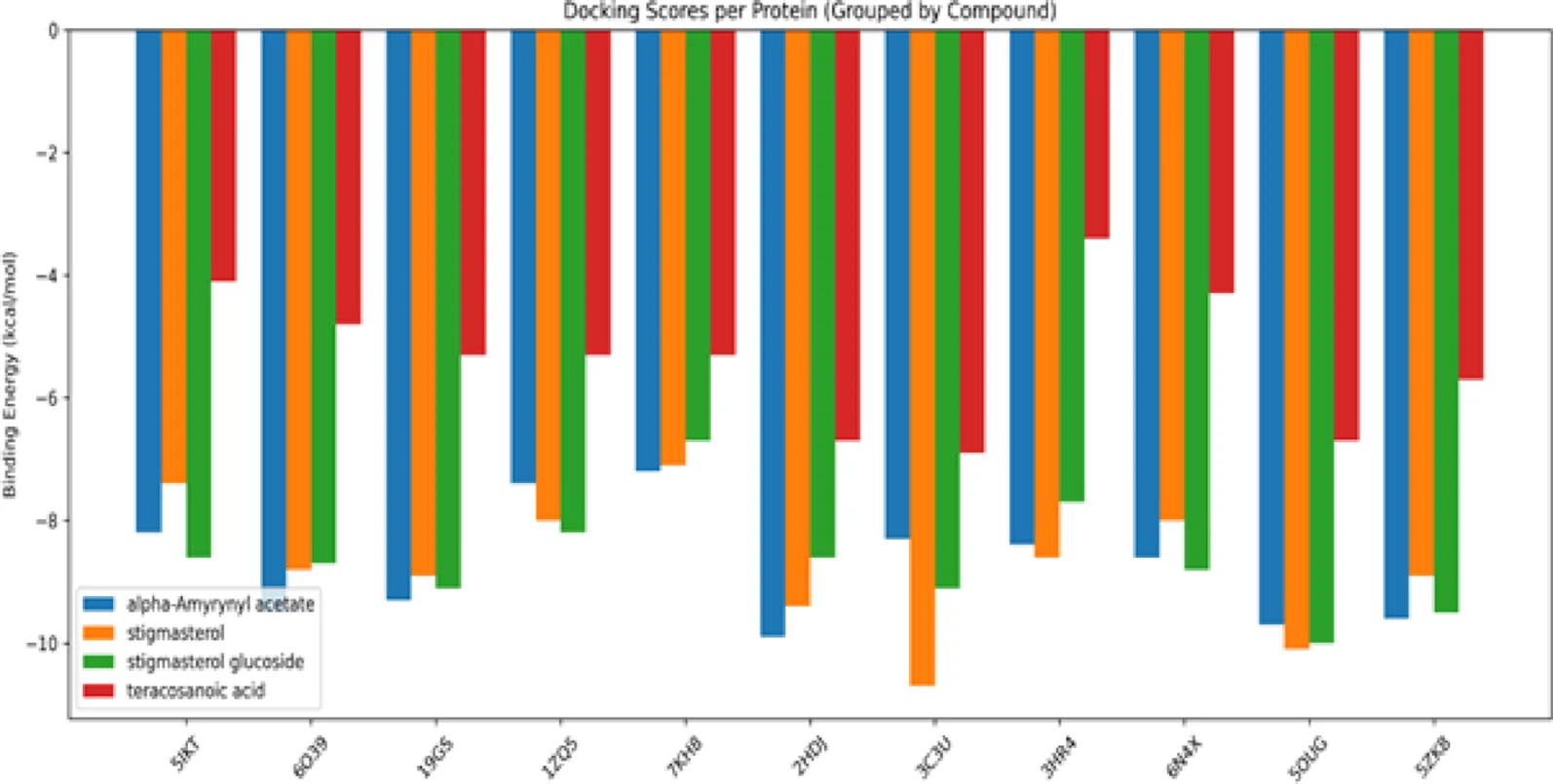
Comparisons of docking scores with Phytocompounds

KEGG pathway enrichment analysis further supports the multitarget and multi pathway nature of the phytocompounds by shedding light on the possible biological pathways through which the predicted targets might show therapeutic effect, this study pointed out several signaling pathways linked to immune regulation, inflammation and host-pathogen interactions. Among those enriched pathways the PI3K-Akt signaling pathway is pivotal in controlling cell survival, immune signaling and the invasion of intracellular pathogen whereas the MAPK signaling pathway is a significant mediator of inflammatory responses and cytokine production during bacterial infections and the TNF signaling pathway is another crucial immune pathway that plays a role in managing inflammatory response and host defense mechanism activation of this pathway signal cytokine release and active immune cells that important manage bacterial infections, from all those enriched pathways calcium signaling (Figure S2) and chemical carcinogenesis -ROS pathways showed highest number of hub targets suggesting potential role in mediating the predicted therapeutic effects and other enriched pathways as arachidonic acid xenobiotic metabolism by cytochrome P450. Steroid hormone biosynthesis is linked to inflammatory mediators and metabolic responses during infections. Molecular docking is a computational technique(Gopi et al. 2025) to predict the best or preferred orientation and binding affinity also interactive residues of ligand against receptor molecules forming a stable complex used as most common structured-based drug design, allowing identifying new drug candidates by screening large virtual libraries of compounds (Patel & Kayande; Rahmatullah 2021). Research has shown that essential oil compounds derived from medicinal plants possess antimicrobial properties and molecular docking has been employed to elucidate their interaction with microbial targets (Roy et al. 2024) whereas, our molecular docking experiment with 4 phytocompounds of *H. javanica* against 11target receptor molecules viz. 5IKT6,O39,19GS, 1ZQ5, 7KH8, 2HDJ, 3C3U, 3HR4, 6N4X, 5OUG, 5ZK8 showing distinct ranging scores of binding affinities (Fig. 16) from highest binding affinity showed by stigmasterol (-10.7 kcal/mol) to lowest binding affinity by tetracosanoic acid (-3.4 kcal/mol) and other two compounds showing moderate binding affinity score over − 5.0 kcal/mol. Stigmasterol showing highest binding affinity against *Staphylococcus aureus* proteins it scored high affinity for DHFR (-9.8 kcal/mol) and sortase A (-8.8 kcal/mol) (Pandit et al. 2024) with inhibitory mechanism such as disrupting bacterial replication and metabolic pathways suggesting potent drug for broad-spectrum inhibitor for different strains (Dahiru et al. 2024; Pandit et al. 2024). Stigmasterol glucoside act as top ranked compound against antibacterial agents, it showing high binding affinity against beta-lactamase of *E.coli* with binding score −10.4 kcal/mol (Swain and Padhy 2015), bacterial protein DNA gyrase a primary target for antibiotics like ciprofloxacin and tetracycline, stigmasterol achieved to show a binding affinity of -7.0 kcal/mol against *E.coli* DNA gyrase B (Gebrehiwot et al. 2024). Overall study stigmasterol and stigmasterol glucoside act as potent antibacterial agents with relevant high binding score and frequently surpassing the docking scores of popular antibiotics particularly against beta-lactamase resistance mechanism (Dahiru et al. 2024). Similar integrative.(Fig. 16)

Approaches combining network pharmacology and molecular docking have been successfully employed to elucidate the therapeutic potential of plant derived compounds, supporting reliability of such compounds(Sowmya et al. 2026). MD simulations act as “computational microscope”, with atomic level insights into the structure, function, and dynamics of biological systems that exceed the capabilities of traditional wet-lab experiments (Gonzalez-Fernandez et al. 2021) (Ting et al. 2021)giving a rational designs of novel therapeutics such as antibiotics and facilitating improve research efficacy, reducing cost through repetitive laboratory assays (Gonzalez-Fernandez et al. 2021) allowing researchers to access biologically meaning full time scales developing safer and more effective clinical treatments. Based on the top scoring result obtained from docking experiment, we conclude that MD simulation for the stigmasterol and 3C3U complex running for 100 ns. MD production. Analyzing the simulation trajectories overall MD simulation indicates that the protein-ligand complex exhibits structural stability through-out the simulation, RMSD shows (Fig. 13A) fluctuations after initial equilibration and smaller and low RMSD values steady and stable structure (Ting et al. 2021), RMSF shows (Fig. 13B) limited flexibility around loop regions and values below 0.2 nm indicating satisfactory or structural stability(Aris et al. 2024), and Radius of gyration (Fig. 13C) remain stable confirming their structural flexibilities, if Rg plots showed no notable variation in the arrangement of the protein when ligands were bound indicating overall structural arrangement remained intact (Aris et al. 2024), H-bond interaction (Fig. 14D) interprets stable binding while the PCA shows (Fig. 14E) restricted conformational movement with clustered trajectories. These trajectories analysis revealed stable and favorable protein ligand interaction dynamics (Harakeh et al. 2023).This retrieved compounds suggesting strong binding affinities and favorable pharmacokinetic properties suggesting its potential as a therapeutic agent against enterobacterial infection. However, the therapeutic efficacy of the bioactive compounds can be further enhanced by advance formulation strategies(Mohammed et al. 2026; Sivasuriyan et al. 2025).

Despite the current in-silico analysis revealed relevant results, certain limitations need to be recognized. The research solely depends on computational predictions, which may not completely capture the complexity of biological systems. Although advance organoid model and organ-on -a-chip systems(Sakthivel et al. 2026) provide more physiologically relevant environments for examining host-pathogen interactions, their use continues to be resource-heavy. Consequently, additional experimental validation is necessary to verify the therapeutic potential of the retrieved compounds.

## Conclusion

The present study on in-silico based comprehensive insight into the possible multi-target potential of retrieved phytocompounds (tetracosanoic acid, alpha-amyrenyl acetate, stigmasterol glucoside and stigmasterol from *H. javanica* against MDR enterobacterial infections. Study on drug-likeness and ADMET screening of compounds evaluates compounds suitability as potential drug candidates with favourable pharmacokinetic properties and bioactivity profiles. Network pharmacology approach identified crucial targets or Hub-targets HSD11B1, PTGS, AKR1C2 and relevant pathways highlighting calcium signaling pathway and emphasizing their critical role disease development and progression. While molecular docking and MD simulation indicated stable interactions between ligands and protein receptor stigmasterol. The highest binding affinity of stigmasterol (-10.7 kcal/mol) against 3C3U target protein reveal stable binding behaviour and interaction efficacy. This study concluded that this is the first time in-silico comprehensive study of *H. javanica* to explore antibacterial potentiality. Out of four phytocompounds of *H. javanica* stigmasterol have potential therapeutic activity to treat enterobacterial infection and in future it became a novel anti-enteric drug formulation for human endeavour. However, despite the promising therapeutic potential suggested by these findings additional in-vivo validation studies are required to validate the therapeutic potential of these phytocompounds. Collectively, this work provides a robust computational framework for plant-based anti-enteric drug discovery and highlights the growing relevance of anti-enteric drug discovery and explores the growing relevance of integrative in-silico strategies in accelerating the identification of novel therapeutics.

## Supplementary Information

Below is the link to the electronic supplementary material.

Supplementary Material 1

## Data Availability

The datasets used and or analysed during the current study are available from the corresponding author on reasonable request.
