# Supplementary material for: In-silico study of active phytochemicals and molecular mechanism of Hydrocotyle javanica Thunb.in treating MDR enterobacterial infection
Source: Bioresour Bioprocess. 2026 Aug 20;13(1):121. doi: 10.1186/s40643-026-01078-5 (PMC13493727; doi:10.1186/s40643-026-01078-5)
Supplement: Supplementary file 1 [file 40643_2026_1078_MOESM1_ESM.docx]

**In-silico study of Active Phytochemicals and Molecular Mechanism of	*Hydrocotyle javanica* Thunb. in Treating MDR Enterobacterial	infection**

Debasmita Paul^1^, Megha Ghosh^2^, Manab Mandal^2^*

^1^ Department of Botany, Jogamaya Devi College, Kolkata, West Bengal- 700026, India.

^2^ Department of Botany, Dukhulal Nibaran Chandra College, Murshidabad-742201, India.

**Corresponding author:** Dr. Manab Mandal, Assistant Professor, Department of Botany, Dukhulal Nibaran Chandra College, Murshidabad-742201, India,

Email Id: [manabmandal130@gmail.com](mailto:manabmandal130@gmail.com)

ORCID ID: **0000-0002-9934-3235**

**Table S1**: Compounds hierarchical classification.

| InChIKey | Kingdom | Superclass | Class | Subclass | Parent Level 1 |
| --- | --- | --- | --- | --- | --- |
| QZZGJDVWLFXDLK-UHFFFAOYSA-N | Organic compounds | Lipids and lipid-like molecules | Fatty Acyls | Fatty acids and conjugates | Very long-chain fatty acids |
| UDXDFWBZZQHDRO-NSTYLNEOSA-N | Organic compounds | Lipids and lipid-like molecules | Prenol lipids | Triterpenoids | Triterpenoids |
| VWDLOXMZIGUBKM-AUGXRQBFSA-N | Organic compounds | Lipids and lipid-like molecules | Steroids and steroid derivatives | Stigmastanes and derivatives | Stigmastanes and derivatives |
| HCXVJBMSMIARIN-PHZDYDNGSA-N | Organic compounds | Lipids and lipid-like molecules | Steroids and steroid derivatives | Stigmastanes and derivatives | Stigmastanes and derivatives |

**Figure S1**: Diagram illustrating the comparative distribution of Enterobacterial infection associated targets retrieved from the Comparative Toxicogenomics Database (CTD) and GeneCards. The larger circle in yellow representing the total disease associated targets retrieve from CTD and smaller circle in green color representing disease associated targets retrieve from GeneCards.

**Figure S2**: Calcium signalling pathway.

**Figure S3**: GO analysis bar plot.
